# Two circPPFIA1s negatively regulate liver metastasis of colon cancer via miR-155-5p/CDX1 and HuR/RAB36

**DOI:** 10.1186/s12943-022-01667-w

**Published:** 2022-10-12

**Authors:** Haein Ji, Tae Won Kim, Woo Joo Lee, Seong Dong Jeong, Yong Beom Cho, Hyeon Ho Kim

**Affiliations:** 1 Department of Health Sciences and Technology, Samsung Advanced Institute for Health Sciences and Technology, Sungkyunkwan University, Seoul, 06351 Republic of Korea; 2 Department of Surgery, Samsung Medical Center, Sungkyunkwan University School of Medicine, Seoul, 06351 Republic of Korea; 3 Research Institute for Future Medicine, Samsung Medical Center, Seoul, 06351 Republic of Korea

**Keywords:** Colorectal cancer, Liver metastasis, circPPFIA1, miR-155-5p, CDX1, HuR, RAB36

## Abstract

**Background:**

Circular RNAs (circRNAs) play a critical role in colorectal cancer (CRC) progression, including metastasis. However, the detailed molecular mechanism is not fully understood.

**Methods:**

Differentially expressed circRNAs between primary KM12C and liver metastatic KM12L4 colon cancer cells were identified by microarray. The expression of circRNAs was measured by semi-quantitative (semi-qPCR) and real time-quantitative PCR (RT-qPCR). Metastatic potential including invasive and migratory abilities, and liver metastasis were examined by transwell assays and intrasplenic injection, respectively. CircPPFIA1-associated microRNA (miRNA) and RNA-binding protein (RBP) were screened by an antisense oligonucleotide (ASO) pulldown experiment. The effects of circPPFIA1 on target gene expression were evaluated by RT-qPCR and western blot analyses.

**Results:**

By analyzing circRNA microarray data, we identified two anti-metastatic circRNAs generated from *PPFIA1* with different length, which named circPPFIA1-L (long) and -S (short). They were significantly downregulated in liver metastatic KM12L4 cells compared to primary KM12C cells. The knockdown of circPPFIA1s in KM12C enhanced metastatic potential and increased liver metastasis. Conversely, overexpression of circPPFIA1s weakened metastatic potential and inhibited liver metastasis. circPPFIA1s were found to function as sponges of oncogenic miR-155-5p and Hu antigen R (HuR) by an ASO pulldown experiment. circPPFIA1s upregulated tumor-suppressing CDX1 expression and conversely downregulated oncogenic RAB36 by decoying miR-155-5p and by sequestering HuR, respectively.

**Conclusion:**

Our findings demonstrate that circPPFIA1s inhibit the liver metastasis of CRC via the miR-155-5p/CDX1 and HuR/RAB36 pathways.

**Supplementary Information:**

The online version contains supplementary material available at 10.1186/s12943-022-01667-w.

## Background

Colorectal cancer (CRC) is the third most common type of malignant tumor and the second leading cause of cancer-related death [1]. Despite advances in treatment, the prognosis of CRC patients is poor, and the mortality rate of CRC continues to rise. The main cause of high mortality is liver metastasis of CRC [2, 3]. Twenty percent of patients with CRC present with metastasis at the time of diagnosis, and approximately 50% eventually develop liver metastasis [4]. Moreover, the overall 5-year survival rate of patients with liver metastasis is only 14% [5].

Recent CRC research on the development of diagnostic and therapeutic targets has gradually expanded from protein-coding genes to non-coding RNAs, such as microRNAs (miRNAs), antisense transcripts, long intergenic non-coding RNAs, and circular RNAs (circRNAs) [6, 7]. Although they are recognized as splicing error, circRNAs have been actively investigated as a means of controlling gene expression [8]. CircRNAs are predominantly generated by back-splicing and are characterized by a covalently-closed loop structure without a 5’ cap and 3’ poly-A tail [9]. Due to their special structure, circRNAs have a higher tolerance to exonucleases, making them remarkably stable. Although their biogenesis is largely unknown, circRNAs are a powerful tool in the diagnosis and treatment of cancer [10–12].

Several circRNAs are reported to influence metastatic potential by acting as sponges of microRNA (miRNA) and RNA-binding protein (RBP) in CRC [13–15]. For example, two miR-145-5p-sponging circRNAs, circRUNX1 and circPVT1, promote CRC metastasis by upregulating target expression [16, 17]. Circ_0001178 enhances metastatic potential of CRC by hindering *ZEB1*-targeting miR-382/587/616 [18]. Conversely, circITGA7 inhibits the lymphatic metastasis of CRC through miR-370-3p/NF1 [19].

Here, we aimed to identify novel circRNAs that can control the liver metastasis of CRC. Through a circRNA microarray, two circPPFIA1s were found to be downregulated in liver metastatic CRC. Transwell assays and intrasplenic injection mouse experiments revealed that circPPFIA1s negatively regulated metastatic potential and liver metastasis of CRC. Furthermore, we found that circPPFIA1s exhibited anti-metastatic effects by sponging oncogenic miR-155-5p, thereby increasing caudal type homeobox 1 (CDX1) expression, and decreasing the expression of RAB36 via the sequestration of an oncogenic RBP, Hu antigen R (HuR). Taken together, we demonstrated that circPPFIA1s are promising therapeutic targets for treatment for liver metastatic CRC.

## Methods

### Clinical specimens

All tissues were collected from CRC patients who had undergone surgery at Samsung Medical Center (Seoul, Korea). Six pairs of primary CRC tumor tissues and corresponding liver metastatic tumor tissues were obtained from surgical resections of CRC patients without any radiotherapy or chemotherapy before surgery. The samples were pathologically confirmed and stored in liquid nitrogen after surgery until use. All human specimens were approved by the Institutional Review Board of the Samsung Medical Center (IRB approval No. 2010-04-004 and 2019-03-054). Written informed consent was obtained from all patients.

### In situ hybridization

The expression levels of circPPFIA1-L and -S in tissues were assessed by BaseScope Assay (Advanced Cell Diagnostics, Newark, CA, USA). Basescope probes for circPPFIA1-L and -S were designed to target the junction sequences of circPPFIA1-L and -S. BaseScope assays were performed using BaseScope Detection Reagent Kit-RED (ACD, Cat. No. 322,900) in accord with the manufacturer’s protocol. Fast RED followed by counterstaining with hematoxylin (Cancer Diagnostics, Inc. USA). The images were visualized using ScanScope AT turbo (Aperio, CA) and analyzed by ImageScope (Aperio, CA).

### Cell culture and transfection

Primary and liver metastatic CRC cells (KM12C and KM12L4, respectively) and colorectal cancer cells (DLD1 and RKO) were cultured with Dulbecco’s modified Eagle’s medium (Gibco, Grand Island, NY, USA). All cell lines were free of mycoplasma contamination and verified by STR analysis (Supplementary Table S1). Cells were transfected with small interfering RNAs (siRNAs) and miRNAs using Lipofectamine 2000 (Invitrogen, Thermo Fisher Scientific, Waltham, MA, USA) according to the manufacturer’s protocol. Detailed information on transfection was shown in Supplementary methods.

### Microarray analysis

Total RNA was isolated from KM12C and KM12L4 cells using TRIzol reagent (Invitrogen, Thermo Fisher Scientific) as described by the manufacturer’s protocol. The circRNA microarray was performed by Arraystar (Arraystar, Rockville, MD, USA). The differentially expressed circRNAs were analyzed with the criteria of *p* < 0.05 and fold-change > 2.0 (Supplementary Figure S2).

### Characterization of circRNA

The circular structure of circPPFIA1-L and -S was confirmed by testing the stability via RNase R resistance and actinomycin D treatment. To verify the divergent region of circPPFIA1-L and -S, Sanger sequencing was carried out. Detailed information on experimental procedures was shown in Supplementary methods.

### Ribonucleoprotein immunoprecipitation (RIP)

The RIP assay was performed using Dynabeads^®^ Protein G (Thermo Fisher Scientific) as described in a previous report [20]. Briefly, the beads were coated with IgG or the indicated antibody (Ago2 antibody, Sigma, St. Louis, MO, USA or HuR antibody, Santa Cruz Biotechnology, Dallas, TX, USA). After equal amounts of PEB lysate were incubated with antibody-coated Dynabeads for 4 h, the beads were washed several times with NT2 buffer (Supplementary Table 4). Following treatment with DNase I (Ambion) and protease K (Bioneer), RNA was isolated by precipitation with absolute ethanol. The level of mRNA in RIP was quantified by RT-qPCR.

### Antisense oligonucleotide (ASO) pull-down assay

To identify circPPFIA1-associated miRNAs and RBPs, an ASO pull-down assay was performed using non-overlapping biotinylated ASOs recognizing the convergent region of each circPPFIA1. Three and two ASOs were prepared for circPPFIA1-L and -S, respectively (Supplementary Figure S14). PEB lysates were incubated with 1 µg of biotinylated ASOs at 4 °C for 2 h. After incubation, 40 µl of pre-washed streptavidin-coupled Dynabeads^™^ (Invitrogen) were added for 4 h at 4 °C. LacZ ASO was used as a negative pulldown control (Supplementary Figure S15C). The RNA was isolated from the pull-down materials using TRIzol, and RT-qPCR or western blot analysis was performed to check the level of miRNA or RBP, respectively.

### Determination of in vivo liver metastasis by intrasplenic injection

Six- to seven-week-old female BALB/c nude mice (Orient Bio, South Korea) were anesthetized with a mixture of ketamine (#7001, Seoul, South Korea) (30 mg/kg) and xylazine (Rompun^®^, Bayer, Leverkusen, Germany) (10 mg/kg) via intraperitoneal injection. A small left abdominal flank incision was made, and the spleen was exteriorized for intrasplenic injection. For the preparation of the cells to be injected, KM12C and KM12L4 cells were transfected with circPPFIA1 siRNA or the overexpression vector, respectively. An equal number of transfected cells (2.0 × 10^6^ cells) were suspended in 50 µl of Hanks’ Balanced Salt Solution (Gibco) and injected into mouse spleens with a 30-gauge needle. After 4 weeks, we examined the liver metastasis with magnetic resonance imaging (MRI) and sacrificed the mice to obtain liver tissues. The animal experiments were performed in a specific pathogen-free animal experiment center at the Samsung Medical Center. Ethics approval for animal use was obtained from the Samsung Medical Center Laboratory Animals Committee (approval number: 20,200,410,002).

## Results

### circPPFIA1s were downregulated in liver metastatic colon cancer cells

**Fig. 1:**
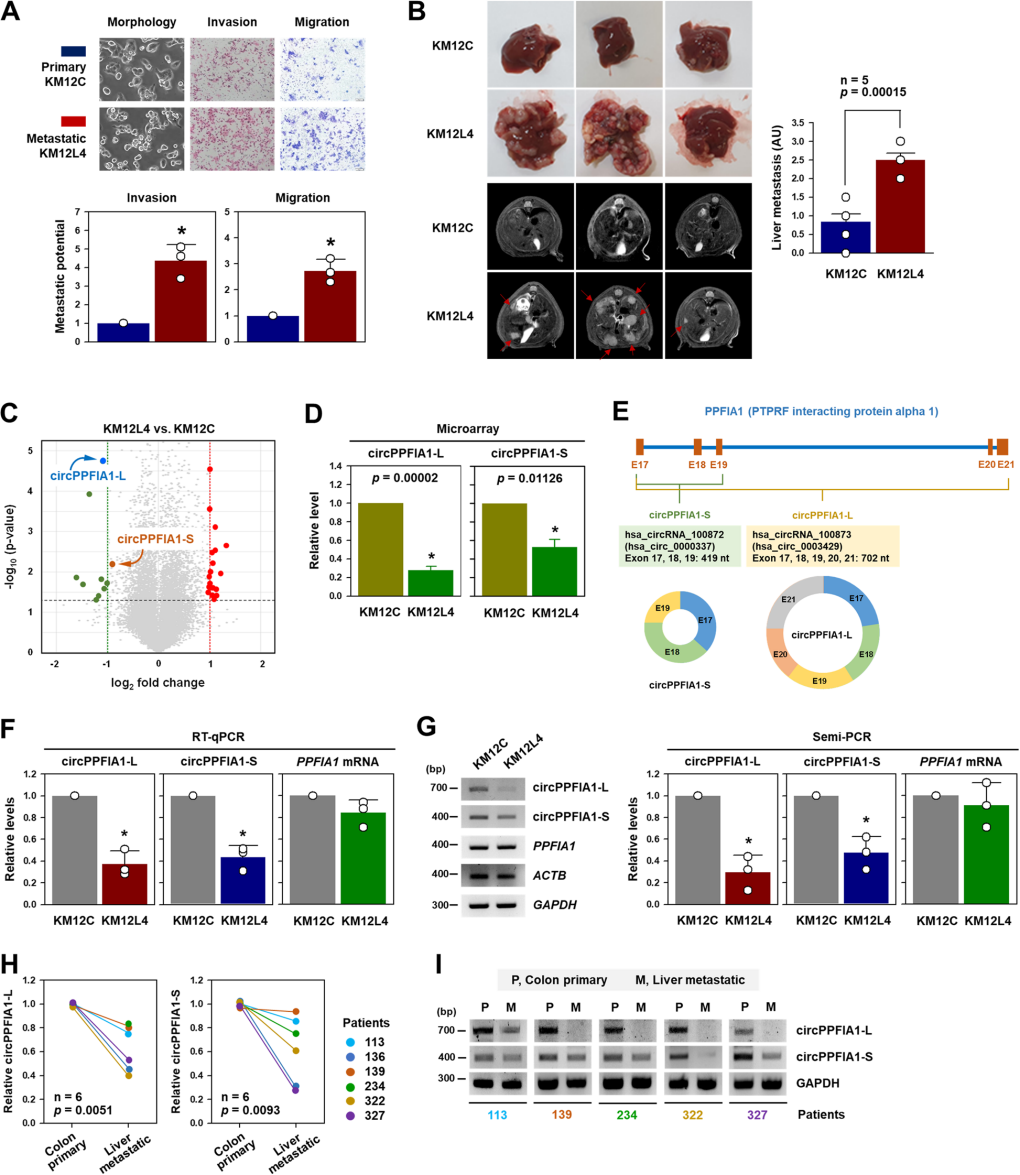
circPPFIA1 is downregulated in liver metastatic colorectal cancer. **A** Transwell invasion and migration assays were carried out to examine the invasive and migratory abilities of KM12C and KM12L4 cells. Metastatic potential was determined by counting the number of invaded and migrated cells, and the representative images are shown. **B** Liver metastasis was examined through in vivo intrasplenic injection. At four weeks post-injection, the optical and MRI images were obtained. The degree of liver metastasis (*n* = 5) was calculated by giving scores in arbitrary units (0–3). **C** Volcano plot illustrating differentially expressed circRNAs in KM12C versus KM12L4. Blue and red dots represent circPPFIA1-L and -S, respectively. **D** Expression level of circPPFIA1-L and -S in KM12C and KM12L4 cells. **E** Schematic illustration and detailed information of circPPFIA1-L and -S. **F**, **G** Expression levels of circPPFIA1-L, -S, and linear PPFIA1 mRNA were determined by RT-qPCR (F) and semi-qPCR (G). **H**, **I **The levels of circPPFIA1-L and -S in primary and liver metastatic colon cancer tissues were determined by RT-qPCR (H) and semi-qPCR (I). Statistical significance was calculated from three independent experiments using the Student’s *t*-test (*p < 0.05). All data represent mean ± standard deviation (SD)

To search for metastasis-associated circRNAs, a previously established cell line model was used: primary colon cancer KM12C cells and its liver metastatic derivative KM12L4 cells (Supplementary Figure S1A) [21]. The high metastatic potential of KM12L4 cells was verified by comparing their invasive and migratory abilities with those of parental KM12C cells. Transwell invasion and migration assays revealed that KM12L4 cells showed a higher number of invaded and migrated cells compared to KM12C cells, indicating that KM12L4 cells have a higher metastatic potential than KM12C cells (Fig. 1 A). To examine the degree of liver metastasis in vivo, KM12C and KM12L4 cells were injected into the spleen and the degree of liver metastasis was determined by visual counting and MRI. As expected, more liver metastases were found in mice injected with KM12L4 cells than in those injected with KM12C cells (Fig. 1B; Supplementary Figure S1B).

A circRNA microarray was conducted to identify differentially expressed circRNAs between KM12C and KM12L4 cells. Twenty-nine circRNAs were differentially expressed more than two-fold. Nine circRNAs showed decreased expression in KM12L4 cells compared to KM12C cells. In contrast, the expression of 20 circRNAs was increased (Supplementary Figure S2A, B). Among them, hsa_circRNA_100873 (hsa_circ_0003429) showed the most significant decrease in expression. hsa_circRNA_100873 is an exonic circRNA generated from five exons (exons 17–21) of PTPRF interacting protein alpha 1 (*PPFIA1*) (Fig. 1E). Interestingly, another *PPFIA1*-originated circRNA, hsa_circRNA_100872 (hsa_circ_0000337), was found in the list of differentially expressed circRNA (Fig. 1C,D). The spliced length of hsa_circRNA_100872 generated from three exons (exons 17–19, 419 bp) is shorter than that of hsa_circRNA_100873 (702 bp). Hence, we named them circPPFIA1-L (long) and circPPFIA1-S (short), respectively (Fig. 1E; Supplementary Figure S2B). According to the circBase database (www.circbase.org), 37 circRNAs are possibly generated from *PPFIA1* (Supplementary Figure S3). However, there are very few circRNAs generated from *PPFIA1* whose action mechanisms and roles have been identified.

To validate the microarray data, the expression levels of circPPFIA1-L and -S were determined by RT-qPCR and semi-qPCR (Supplementary Figure S4A,C). RT-qPCR analyses using specific primers recognizing their divergent region showed a considerable decrease in both circPPFIA1-L and -S in KM12L4 cells (Fig. 1F; Supplementary Figure S4B). However, linear *PPFIA1* mRNA levels were almost same. Similarly, semi-qPCR analyses revealed that KM12L4 expressed less of both circRNAs compared to KM12C cells without a significant change in linear mRNA levels (Fig. 1G). Similar to the results in the model cell lines, the expression of circPPFIA1 in the tissues of colon cancer patients showed a decrease compared to normal tissues, although significant results were not obtained due to the small sample size (Supplementary Figure S5). In addition, we also checked the expression levels of circPPFIA1-L and -S in primary colon cancer tissues and matched liver metastatic colon cancer tissues by RT-qPCR (Fig. 1H) and semi-PCR (Fig. 1I). Both PCR analyses showed a significant decrease in circPPFIA1-L and -S in liver metastatic tissues.

### circPPFIA1-L and -S are highly stable circularized RNAs

**Fig. 2:**
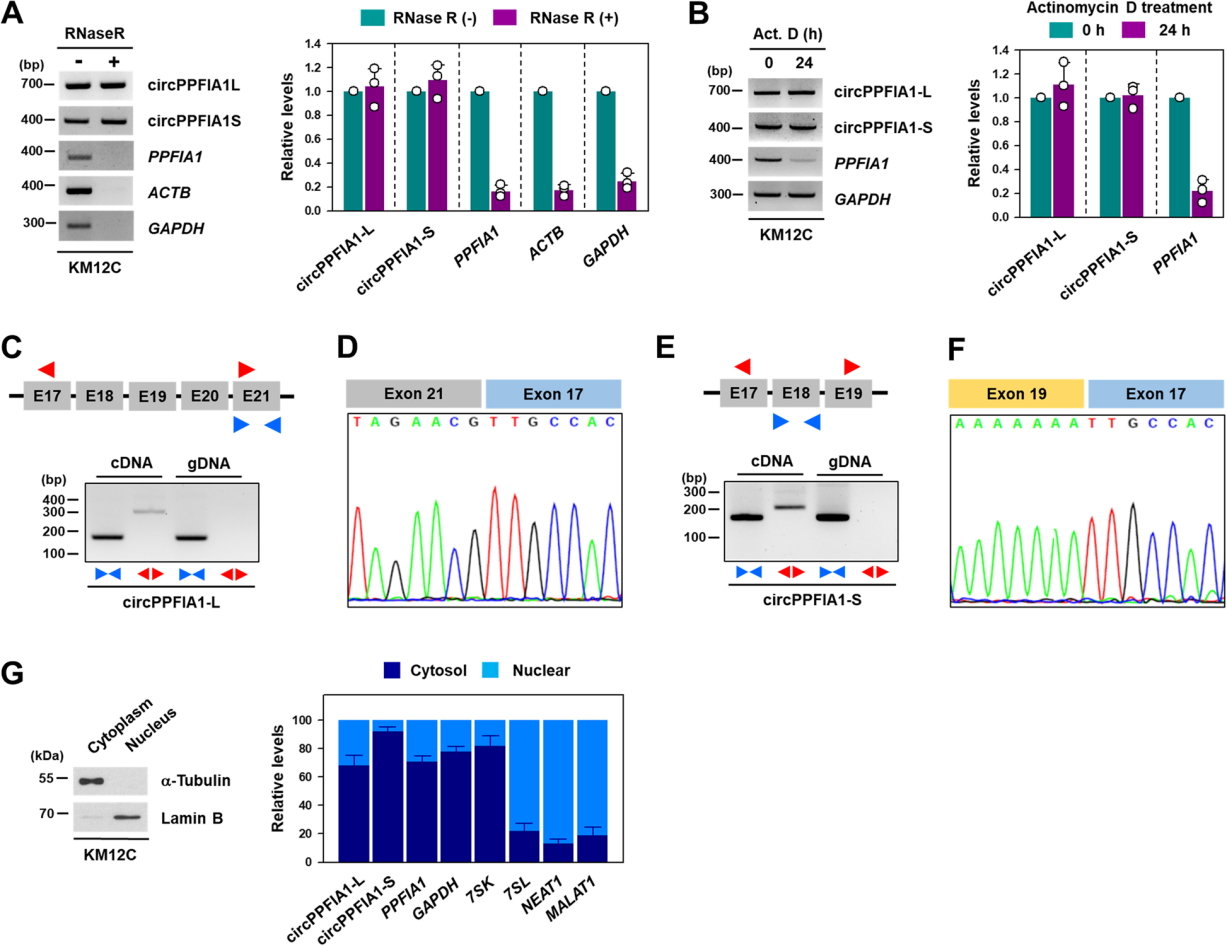
Characterization of circPPFIA1-L and -S. **A**, **B** The stability of circPPFIA1-L and -S was examined by RNase R resistance (**A**) and actinomycin D experiments (**B**). The level of remaining circPPFIA1-L and -S was determined by semi-qPCR and RT-qPCR. Linear *PPFIA1* mRNA, *GAPDH*, and *ACTB* were included as controls. **C**, **E** Complementary (cDNA) and genomic DNA (gDNA) were prepared using RNA isolated from KM12C cells. The level of circPPFIA1-L (**C**) and -S (**E**) was determined by semi-qPCR. **D**, **F** To verify the divergent region of circPPFIA1-L (**D**) and -S (**F**), Sanger sequencing was performed. (**G**) To determine the cellular localization of circPPFIA1s, the fractionation experiment was performed using digitonin. The level of a-Tubulin and lamin B were checked for verification of cytoplasmic and nuclear extracts, respectively. The levels of circPPFIA1-L, -S, and linear *PPFIA1* mRNA were determined by RT-qPCR. *GAPDH* mRNA and *7SK* were used as a marker of cytoplasmic RNA and *NEAT1*, *MALAT1*, and *7SL* were used for nuclear RNA. Statistical analyses were performed using the Student’s *t*-test using three independent experiments (*p < 0.05). All data represent mean ± standard variation (SD)

The stability of circPPFIA1s was assessed by semi-qPCR or RT-qPCR after RNase R and actinomycin D treatment. Whereas RNase R degraded linear *PPFIA1* mRNA, circPPFIA1-L and -S were highly resistant to RNase R (Fig. 2A). Additionally, linear *PPFIA1* mRNA was almost degraded at 24 h post-treatment with actinomycin D. However, neither circPPFIA1s was degraded (Fig. 2B; Supplementary Figure S6A). These results indicate that circPPFIA1-L and -S are highly stable, which is a typical property of circRNAs.

To verify the junction sequence of circPPFIA1-L and -S, genomic DNA (gDNA) and cDNA were used for the PCR analysis with convergent and divergent primers. Whereas PCR products of the convergent primers were observed for gDNA and cDNA templates, the divergent primers generated PCR products only from cDNA (Fig. 2 C for circPPFIA1-L, 2E for circPPFIA1-S). In cDNA and gDNA, *GAPDH* was amplified only by the convergent primer (Supplementary Figure S6B). The back-spliced junction was amplified and verified by Sanger sequencing (Supplementary Figure S4D). We observed head-to-tail splicing between exons 17 and 21 in circPPFIA1-L and exons 17 and 19 in circPPFIA1-S, indicating that they have a circularized structure (Fig. 2D, F; Supplementary Figure S6C).

To assess the localization of circRNA, a cellular fractionation assay was conducted. The level of α-tubulin and lamin B was determined to verify appropriate fractionation. Both circPPFIA1-L and -S were abundantly expressed in the cytosol (Fig. 2G), which suggests that circPPFIA1s could function as molecular sponges of miRNA or RBP.

### circPPFIA1s negatively regulated metastatic potential and liver metastasis of CRC

**Fig. 3:**
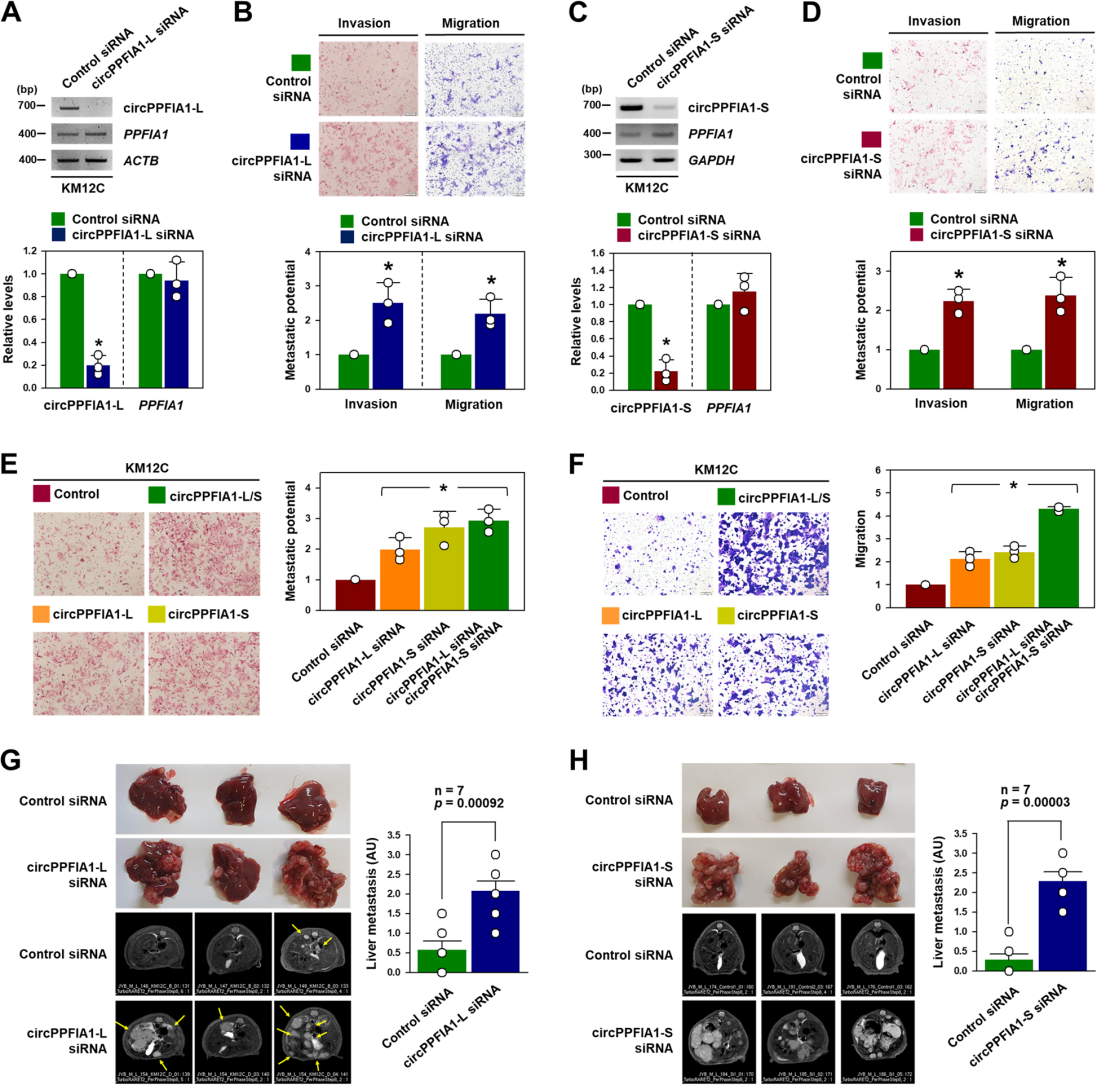
Knockdown of circPPFIA1s enhances the metastatic potential of KM12C cells. **A–D** The effect of circPPFIA1-L and -S silencing on the metastatic potential of KM12C cells was examined. The level of circPPFIA1-L (**A**) and -S (**C**) in KM12C cells transfected with the indicated siRNA was determined by semi-qPCR (upper) and RT-qPCR (lower). Invasive and migratory abilities of transfected cells (knockdown of circPPFIA1-L (**B**) and -S (**D**)) were examined by transwell invasion and migration assays. **E**, **F **The increase in invasive and migratory abilities with knockdown of both circPPFIA1-L and circPPFIA1-S was determined by transwell invasion (**E**) and migration (**F**) assays. **G**, **H** Intrasplenic injection model (*n* = 7) confirmed that knockdown of circPPFIA1-L (**G**) and -S (**H**) significantly promoted liver metastasis of KM12C cells. Statistical analyses were performed using the Student’s *t*-test using three independent experiments (*p < 0.05). All data represent mean ± standard variation (SD)

To investigate whether the knockdown of circPPFIA1-L and -S regulated the metastatic potential of KM12C cells, we designed siRNAs targeting the divergent junctions of circPPFIA1-L and -S (Supplementary Figure S7A for circPPFIA1-L and S7C for circPPFIA1-S). All designed siRNAs showed an efficient decrease in corresponding circRNAs, but barely influenced the expression of linear *PPFIA1* (Fig. 3 A, C; Supplementary Figure S7B and D). An increase in invasive and migratory abilities was observed in circPPFIA1-L- and circPPFIA1-S-silenced KM12C cells (Fig. 3B and D). The increased metastatic potential was observed with each siRNA. However, the knockdown of *PPFIA1* mRNA did not influence the metastatic potential of KM12C cells (Supplementary Figure S8A,B). To exclude the possibility that the increased number of invaded and migrated cells observed after circPPFIA1s knockdown is attributed to increased cell growth, we examined the proliferation rate of circPPFIA1s-silenced KM12C cells. Neither circPPFIA1-L nor circPFIA1-S affected cell growth (Supplementary Figure S8C), demonstrating that circPPFIA1s inhibit the metastatic potential of KM12C cells without affecting cell growth. Notably, metastatic properties, including invasion and migration, were further increased when both circPPFIA1-L and -S were simultaneously silenced (Fig. 3E, F; Supplementary Figure S7E).

To investigate whether knockdown of circPPFIA1-L and -S increased the liver metastasis of CRC in vivo, a splenic injection mouse model was used. An approximate four-fold increase in liver metastasis was observed in the mice injected with circPPFIA1-L-silenced KM12C cells (Fig. 3G; Supplementary Figure S9A, B). The intrasplenic injection of circPPFIA1-S-silenced KM12C cells showed a more than four-fold increase in liver metastasis (Fig. 3 H; Supplementary Figure S9C, D). Based on these results, we confirmed that the knockdown of both circPPFIA1-L and -S potentiates metastatic potential and enhances the liver metastasis of CRC.

**Fig. 4:**
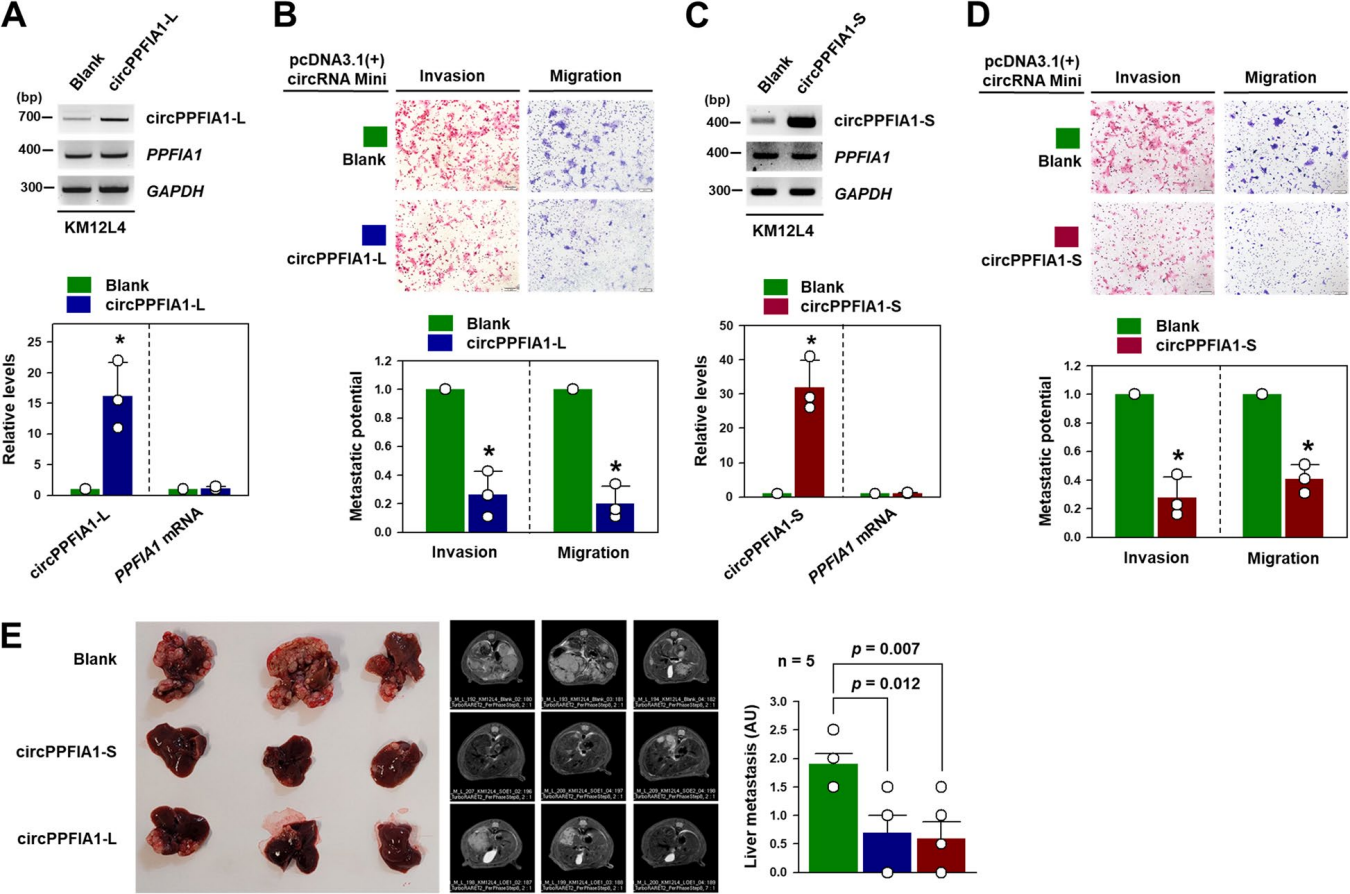
Overexpression of circPPFIA1s attenuates metastatic potential of KM12L4 cells. **A**–**D** To investigate the roles of circPPFIA1s overexpression on the metastatic potential of KM12L4 cells, overexpression vectors for circPPFIA1-L and -S were introduced into KM12L4 cells. The relative levels of circPPFIA1-L (**A**) and -S (**C**) were determined by semi-qPCR (upper) and RT-qPCR (lower). Transwell invasion and migration assays showed that overexpression of circPPFIA1-L (**B**) and -S (**D**) significantly reduced the invasive and migratory abilities of KM12L4 cells. **E** Intrasplenic injection model (*n* = 5) confirmed that overexpression of circPPFIA1-L and -S significantly reduced liver metastasis of KM12L4 cells. Statistical analyses were performed using Student’s *t*-test using three independent experiments (*p < 0.05). All data represent mean ± standard variation (SD)

To examine whether the circPPFIA1s suppressed the metastatic potential of KM12L4 cells, we constructed overexpression vectors expressing each circRNA. Both vectors induced a significant increase in circPPFIA1-L and -S without any change in the linear *PPFIA1* mRNA (Fig. 4 A, C). Increased expression of the circPPFIA1s resulted in the inhibition of the invasive and migratory properties of KM12L4 cells (Fig. 4B and D). Overexpression of circPPFIA1-L dose-dependently suppressed the invasive ability (Supplementary Figure S10A), and similar results were obtained in all overexpressing cells (Supplementary Figure S10B–D). We also confirmed that the reduction in metastatic abilities did not result from the inhibition of cell growth (Supplementary Figure S10E). The intrasplenic injection experiments revealed that KM12L4 cells with high levels of circPPFIA1s showed a decrease in liver metastasis (Fig. 4E). The incidence of liver metastasis and the number of nodules were decreased in mice injected with circPPFIA1-overexpressing KM12L4 cells (Supplementary Figure S11).

To confirm that the inhibitory effects of circPPFIA1s on metastatic properties can be applied to other colon cancer cells, DLD1 and RKO colon cancer cells were used. Similar to the results in KM12C cells, the knockdown of circPPFIA1-L or -S caused an increase in metastatic abilities (Supplementary Figure S12A,B). Conversely, the increased expression of either circPPFIA1-L or -S diminished the number of invaded and migrated cells (Supplementary Figure S12 C, D). Thus, we demonstrate that circPPFIA1-L and -S negatively regulates liver metastasis in CRC.

### Mir-155-5p was identified as a sponging target of circPPFIA1s

**Fig. 5:**
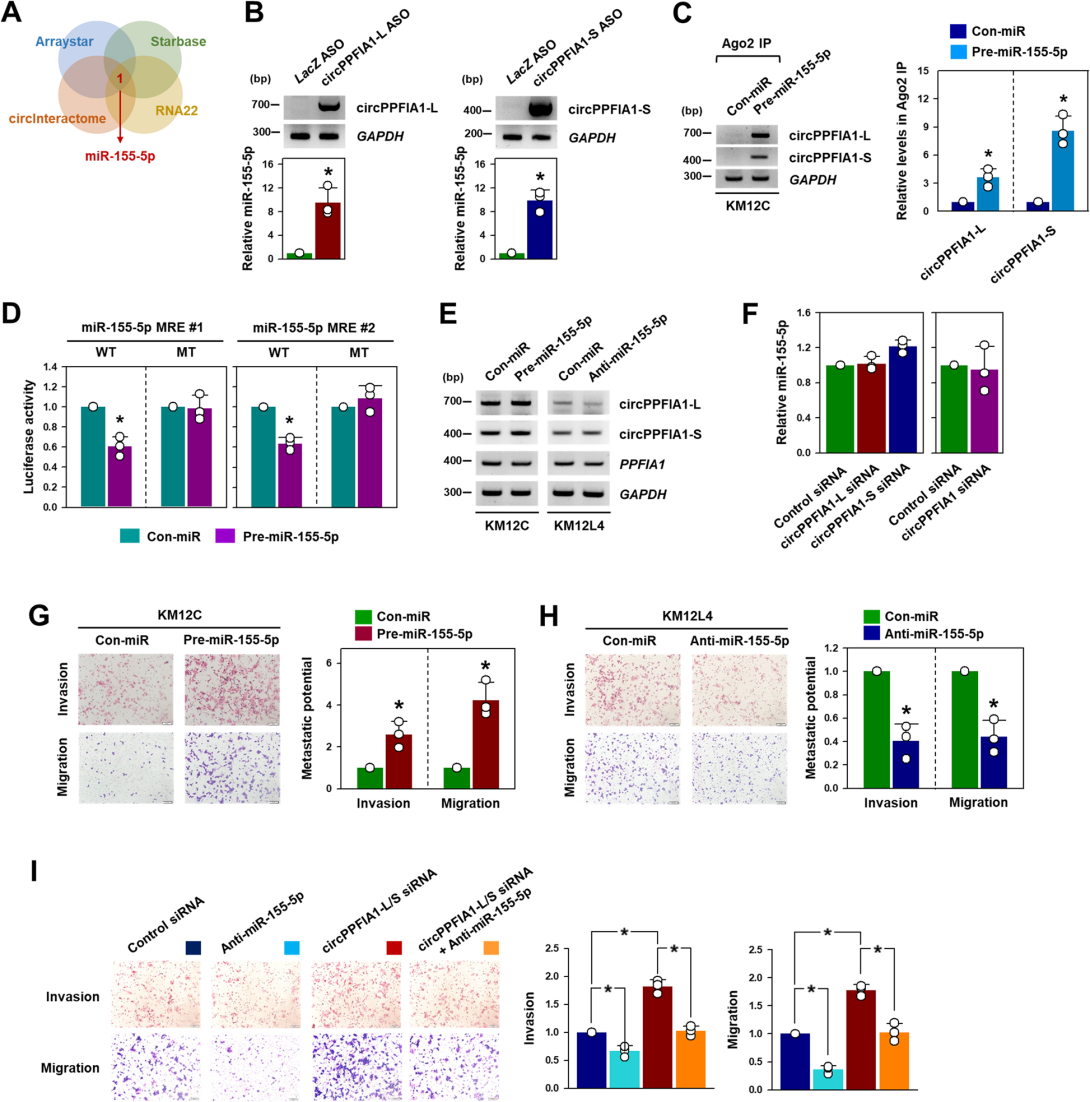
Oncogenic miR-155-5p is a sponging target of circPPFIA1s. **A** Venn diagram showing miR-155-5p predicted as a putative sponging target of circPPFIA1s in four databases (Arraystar, Starbase, circInteractome, and RNA22). **B** To verify that miR-155-5p binds to circPPFIA1s, an ASO pulldown experiment was performed. The pulldown efficacies of circPPFIA1s ASO were examined by semi-qPCR (upper) and the level of miR-155-5p in pulldown materials was checked by RT-qPCR (lower). **C** Enrichment of circPPFIA1-L and -S in miRISC was analyzed by Ago2 RIP with KM12C cells transfected with pre-miR-155-5p. **D** The levels of circPPFIA1-L and -S were determined by semi-qPCR in KM12C and KM12L4 cells transfected with pre-miR-155-5p or anti-miR-155-5p, respectively. **E** To examine the direct interaction between circPPFIA1s and miR-155-5p, the luciferase reporter vectors containing the wild-type or mutant sequence of miR-155-5p MRE were constructed. Following overexpression of miR-155-5p, a luciferase activity assay was carried out in KM12C cells. **F** After transfection of circPPFIA1 siRNAs, the expression level of miR-155-5p was determined by RT-qPCR. **G**, **H** Following transfection of pre-miR-155-5p or anti-miR-155-5p into KM12C (**G**) and KM12L4 (**H**) cells, the Invasive and migratory abilities were measured by transwell invasion and migration assays, respectively. **I** To check whether miR-155-5p is required for increased metastatic potential by knockdown of circPPFIA1s, rescue experiments were conducted. Invasive and migratory abilities were examined by transwell invasion and migrations assays, respectively. Statistical analyses were performed using the Student’s *t*-test using three independent experiments (*p < 0.05). All data represent mean ± standard variation (SD)

Increasing evidence suggests that circRNAs act as miRNA sponges, thereby interrupting the inhibitory functions of miRNA. The cellular fractionation assays revealed that circPPFIA1-L and -S were abundantly located in the cytosol (Fig. 2G), suggesting that they might function as competing endogenous RNAs (ceRNAs). Four bioinformatic prediction algorithms (ArrayStar, https://www.arraystar.com; circInteractome, https://circinteractome.nia.nih.gov; Starbase, http://starbase.sysu.edu.cn; and RNA22, https://cm.jefferson.edu/rna22) were used to search for circPPFIA1-interacting miRNAs. The only common prediction in all algorithms was miR-155-5p (Fig. 5 A; Supplementary Figure S13).

To verify that miR-155-5p interacts with circPPFIA1, an ASO pulldown experiment was performed. First, we designed ASOs targeting the divergent sequences of circPPFIA1-L or -S. All designed ASOs for circPPFIA1-L and -S worked efficiently and miR-155-5p bound to circPPFIA1s (Supplementary Figure S14A, B). Repeated ASO pulldown experiments were conducted by the mixture of corresponding ASOs, and the level of miR-155-5p in pulldown materials was determined by RT-qPCR.

To confirm the interaction between cirPPFIA1s and miR-155-5p, an Argonaute 2 immunoprecipitation (Ago2 IP) experiment was performed. The introduction of pre-miR-155-5p into KM12C cells increased the enrichment of circPPFIA1-L and -S in Ago2 IP material compared to the control IgG (Fig. 5 C). Interestingly, the RT-qPCR results indicated that circPPFIA1-S was more enriched than circPPFIA1-L, assumingly due to the higher cytosolic levels of circPPFIA1-S. In addition, the direct interaction between circRNAs and miR-155-5p was examined by a luciferase assay. Two miR-155-5p MREs were found in exon 18, which is present in both circRNAs (details in Supplementary Figure S15A), and therefore, we constructed two luciferase vectors containing the wild-type or mutated sequence of miR-155-5p MRE (Supplementary Figure S15B). Luciferase activity was inhibited by overexpression of miR-155-5p in both vectors containing wild-type MRE. However, the luciferase expression was not affected in the case of mutated vectors (Fig. 5D).

Although we confirmed that circPPFIA1s and miR-155-5p were bound, the knockdown of circPPFIA1-L, -S, or both did not affect the level of miR-155-5p (Fig. 5E). Overexpression of miR-155-5p by introducing pre-miRNA into KM12C cells did not influence the expression of circPPFIA1s (Fig. 5F). Similarly, the downregulation of miR-155-5p by anti-miRNA in KM12L4 cells did not result in the reduction of circPPFIA1s (Fig. 5F). These results indicate that the circPPFIA1s and miR-155-5p did not affect each other’s expression. The effect of changes in ceRNA on the level of sponging miRNAs has not been fully elucidated. Due to their structural characteristics, circRNAs are not thought to be affected by their sponging miRNAs.

Next, we examined the regulation of metastatic potential by miR-155-5p. The overexpression of miR-155-5p in KM12C cells caused an increase in the number of invaded and migrated cells compared to the control miRNA (Fig. 5G). In contrast, the inhibition of miR-155-5p suppressed the metastatic potential of KM12L4 cells (Fig. 5 H). The regulatory effect of miR-155-5p was confirmed in DLD1 and RKO cells. As observed in KM12C and KM12L4 cells, the metastatic potential was increased depending on the expression level of miR-155-5p (Supplementary Figure S16A for DLD1 and S16A for RKO). To verify the role of miR-155-5p in circPPFIA1s-mediated regulation of metastatic potential, a rescue experiment was conducted using a mixture of siRNAs targeting circPPFIA1-L and -S. As expected, the metastatic potential of KM12C cells was potentiated by the knockdown of circPPFIA1s. However, the inhibition of miR-155-5p reversed the increase in the invasive and migratory abilities of KM12C cells, indicating that an increase in liberated miR-155-5p is responsible for the function of circPPFIA1s (Fig. 5I). Rescue experiments using each circPPFIA1-L and -S siRNA also showed similar results (Supplementary Figure S17).

### CDX1 is responsible for the function of circPPFIA1/miR-155-5p

**Fig. 6:**
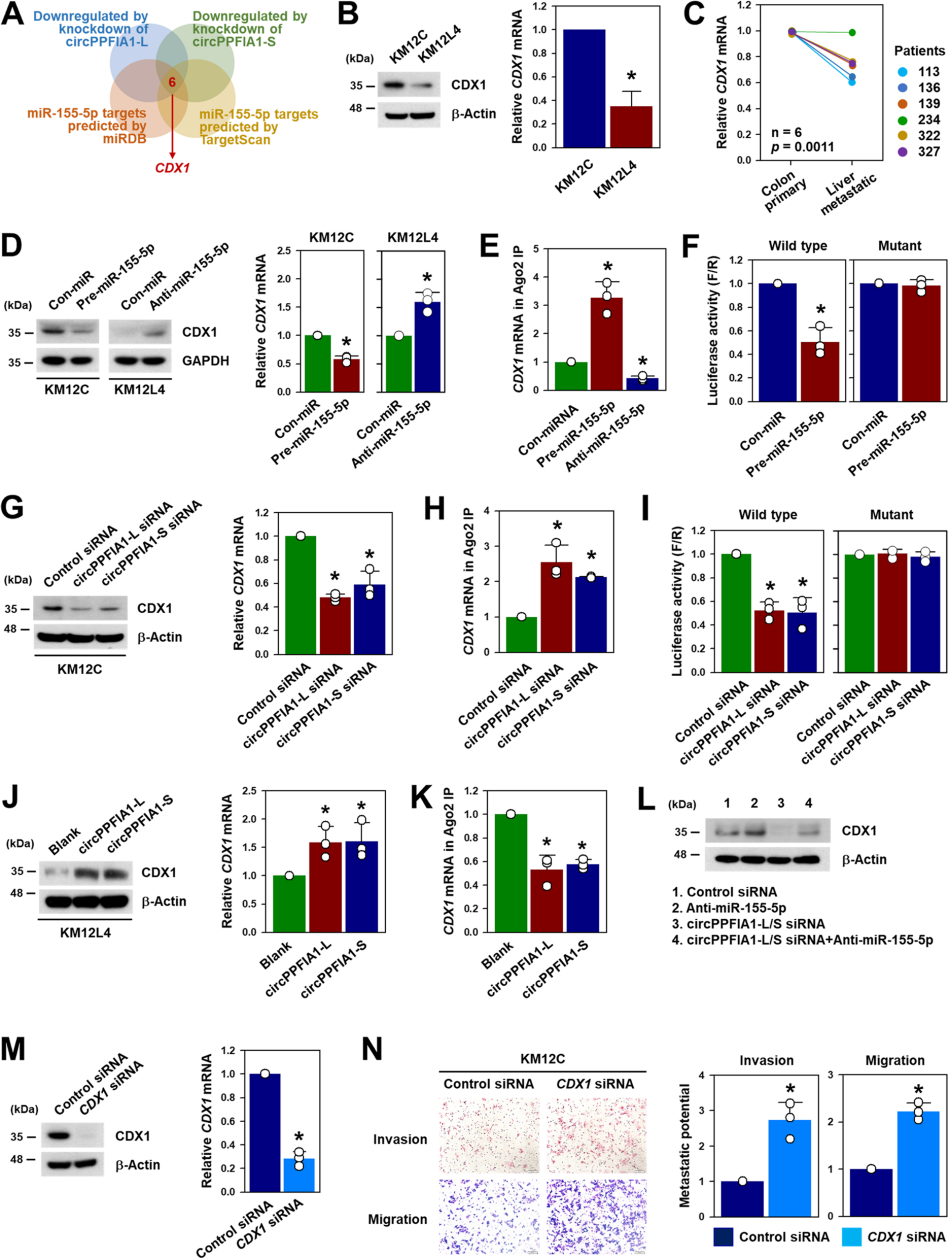
CDX1 is a target of miR-155-5p. **A** *CDX1* is predicted as a target of miR-155-5p by comparing the lists of downregulated mRNAs obtained from RNA sequencing results with databases (miRDB and TargetScan). **B** The expression levels of CDX1 protein and mRNA in KM12C and KM12L4 were compared by western blot and RT-qPCR analyses. **C** The expression level of *CDX1* in primary and liver metastatic colon cancer tissues was determined by RT-qPCR. **D** Following transfection of KM12C and KM12L4 cells with pre-miR-155-5p and anti-miR-155-5p respectively, the levels of CDX1 protein and mRNA were assessed by western blot and RT-qPCR analyses, respectively. **E**, **F** Direct interaction of miR-155-5p with the 3’UTR of *CDX1* mRNA, Ago2 RIP (**E**), and luciferase activity assays (**F**) were performed. **G–I** Following transfection of KM12C cells with siRNAs targeting circPPFIA1-L or circPPFIA1-S, the expression levels of CDX1 protein and mRNA (**G**), the enrichment of CDX1 mRNA in Ago2 RIP (**H**), and luciferase assay (**I**) were performed as described above. **J**, **K** To examine whether overexpression of circPPFIA1s increases CDX1 expression, KM12C cells were transfected with blank or overexpression vectors. The expression levels of CDX1 protein and mRNA were determined by western blot and RT-qPCR, respectively (**J**). The effect of circPPFIA1s overexpression on the interaction between miR-155-5p and *CDX1* mRNA was examined by Ago2 RIP (**K**). **L** To examine whether the inhibition of miR-155-5p restores downregulation of CDX1 by knockdown of circPPFIA1s, western blot analysis was performed. **M**, **N** Following the transfection of KM12C cells with CDX1 siRNA, the efficacy of CDX1 siRNA was examined by western blot and RT-PCR (**M**). The effect of CDX1 silencing on the metastatic potential of KM12C cells was checked by transwell invasion and migration assays (**N**). Statistical analyses were performed using the Student’s *t*-test using three independent experiments (*p < 0.05). All data represent mean ± standard variation (SD)

By screening targets of circPPFIA1/miR-155-5p using prediction algorithms, six genes were identified (Supplementary Figure S18). *CDX1*, a tumor-suppressor, was selected by RT-qPCR validation and reference search for further studies (Fig. 6A). Western blot and RT-qPCR assays revealed that *CDX1* was highly expressed in KM12C cells compared to KM12L4 cells (Fig. 6B). Similarly, the expression level of *CDX1* in liver metastatic colon cancer tissues was lower than that in primary colon cancer tissues (Fig. 6C). The effect of miR-155-5p on the expression of CDX1 was tested using pre- and anti-miR-155-5p in KM12C and KM12L4 cells, respectively. The overexpression of miR-155-5p decreased CDX1 protein and mRNA expression levels in KM12C cells. Conversely, CDX1 was upregulated by decreasing miR-155-5p in KM12L4 cells (Fig. 6D). Direct interaction between *CDX1* mRNA and miR-155-5p was assessed by Ago2 RIP and luciferase experiments. The enrichment of *CDX1* mRNA in Ago2 IP material was enhanced by the overexpression of miR-155-5p and was lowered by the inhibition of miR-155-5p compared to the control (Fig. 6E). One MRE of miR-155-5p in the sequence of the 3’UTR of *CDX1* mRNA was found (Supplementary Figure S19). To confirm the binding of miR-155-5p to *CDX1* mRNA, luciferase vectors containing the wild-type or mutated sequence of the miR-155-5p binding site were manufactured. The overexpression of miR-155-5p significantly decreased luciferase activity; in contrast, the mutation of the binding sequence blocked the miR-155-5p-mediated inhibition of luciferase activity (Fig. 6 F).

We found that circPPFIA1s associated with miR-155-5p and mitigated its inhibitory function. Therefore, we tested whether circPPFIA1s regulated CDX1 expression. The knockdown of circPPFIA1-L or -S decreased the expression level of CDX1 protein and mRNA in KM12C cells (Fig. 6G). Ago2 RIP and luciferase experiments were carried out to verify that miR-155-5p was required for the regulation of CDX1 by circPPFIA1s. The knockdown of circPPFIA1-L and -S increased the enrichment of *CDX1* mRNA in Ago2 IP materials (Fig. 6 H) resulting from an increase in liberated miR-155-5p via a decrease in the level of circPPFIA1 as a ceRNA (Fig. 6 H). Increased levels of functional miR-155-5p due to the knockdown of circPPFIA1-L and -S also lowered luciferase expression in the wild-type but not in the mutant vector (Fig. 6I). These results indicate that the inhibitory effect of miR-155-5p on CDX1 expression is enforced by the knockdown of circPPFIA1s. We also tested whether the overexpression of circPPFIA1s can upregulate CDX1 expression. The expression levels of CDX1 protein and mRNA were increased by the overexpression of circPPFIA1s (Fig. 6J), and as expected, the enrichment of *CDX1* mRNA in Ago2 IP was lowered in circPPFIA1-overexpressing cells (Fig. 6 K).

The above results indicated that the inhibition of miR-155-5p by anti-miR reversed the increased metastatic potential due to the knockdown of circPPFIA1s (Fig. 5I). Accordingly, we assessed the expression level of CDX1 in the same samples. Decreased CDX1 expression due to the knockdown of circPPFIA1s was restored by introducing anti-miR-155-5p into KM12C cells (Fig. 6L). To examine whether CDX1 is associated with metastatic potential, the invasive and migratory abilities of CDX1-silenced KM12C cells were assessed. We found that siRNA that targets *CDX1* mRNA efficiently decreased the expression of CDX1 (Fig. 6 M). Transwell assays revealed that the knockdown of CDX1 enhanced invasive and migratory abilities (Fig. 6 N). These results indicate that liberated miR-155-5p by the knockdown of circPPFIA1s suppressed CDX1 by directly binding to its mRNA.

### HuR is identified as a circPPFIA1s-interacting RBP

**Fig. 7:**
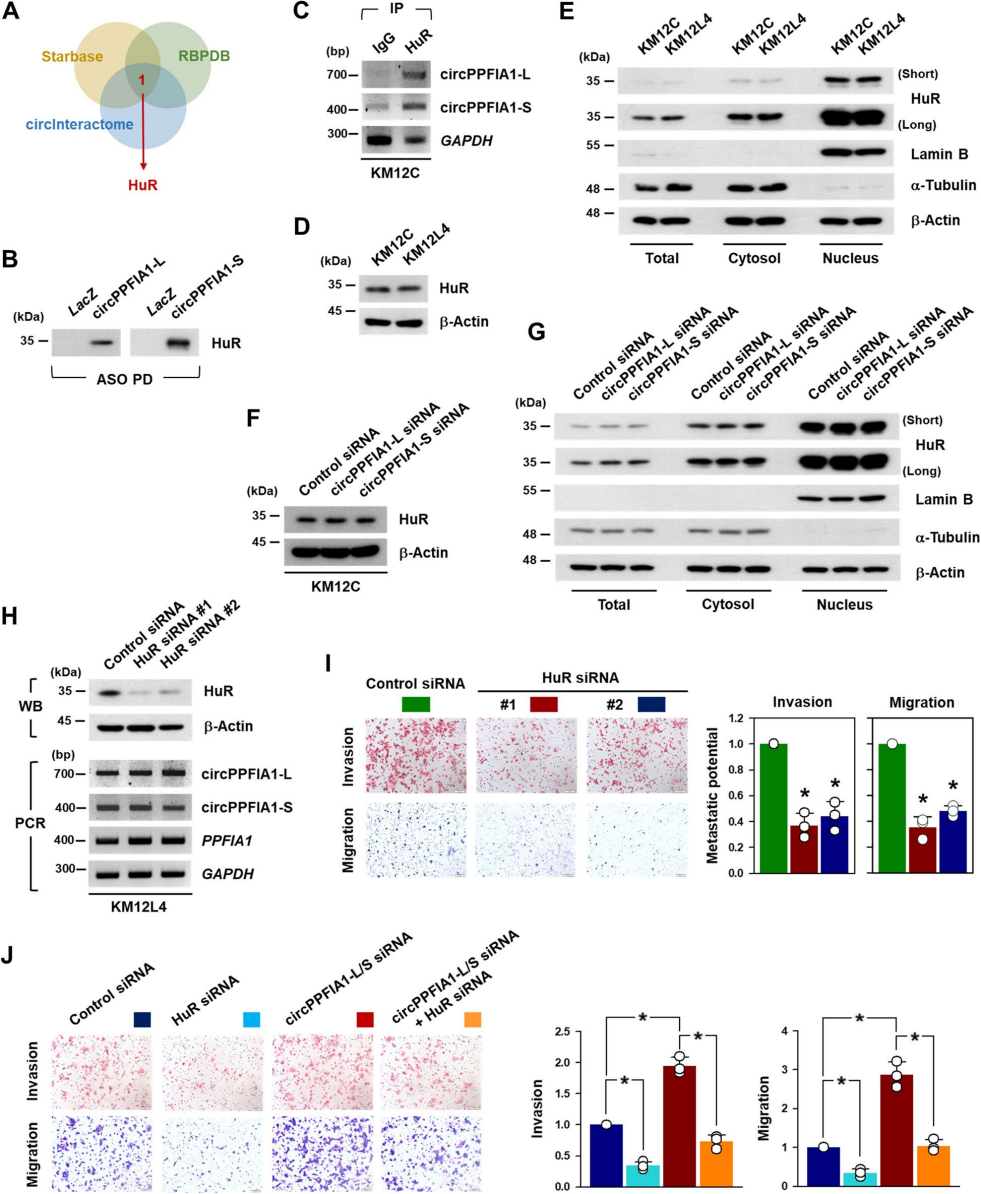
HuR is a sponging target of circPPFIA1s and is required for regulation of metastatic potential. **A** Venn diagram demonstrated the overlapping gene of the interacting RBPs with circPPFIA1 predicted by databases (circInteractome, RBPDB, and Starbase). **B** Validation of interaction of HuR with circPPFIA1s. The level of HuR in ASO pulldown materials was determined by western blot analysis. **C** To demonstrate the interaction between circPPFIA1s and HuR, the level of circPPFIA1-L and -S in HuR RIP was determined by semi-qPCR. **D**, **E** The expression level of HuR in KM12C and KM12L4 cells was determined by western blot analysis (**D**). Localization of HuR was examined by Western blot analysis followed by cellular fractionation (**E**). **F**, **G** The effect of circPPFIA1s silencing on the expression (**F**) and localization (**G**) of HuR was examined by western blot analysis. **H** The expression levels of circPPFIA1-L and -S were determined by semi-qPCR in HuR-silenced KM12L4 cells. **I** Transwell invasion and migration assays were carried out to check whether HuR regulates metastatic potential of KM12L4 cells. **J** Requirement of HuR in circPPFIA1s-regulated metastatic potential. KM12C cells were transfected with indicated siRNAs and the invasive and migratory abilities were examined by transwell invasion and migration assays, respectively. Statistical analyses were performed using the Student’s *t*-test using three independent experiments (*p < 0.05). All data represent mean ± standard variation (SD)

By predicting circPPFIA1s-associated RBPs using three algorithms (circInteractome, RBPDB, and StarBase), HuR was identified as a putative sponging RBP of circPPFIA1s (Fig. 7 A; Supplementary Figure S20A). Moreover, the association of HuR with circPPFIA1s was confirmed by a computational prediction (RBPmap, http://rbpmap.technion.ac.il). To verify the direct interaction between circPPFIA1s and predicted RBPs, ASO pulldown was followed by western blot analysis (Supplementary Figure S20B). Among them, we observed that HuR was significantly bound to both circPPFIA1-L and -S, whereas another RBPs showed weakly or barely bound to circPPFIA1s (Fig. 7B; Supplementary Figure S20C). In addition, the association of HuR with circPPFIA1s was examined by HuR RIP experiments. Semi-qPCR results showed that circPPFIA1s were more enriched in HuR IP than in IgG IP. These results indicate that HuR, as a sponging RBP of circPPFIA1s, is closely implicated in the anti-metastatic function of circPPFIA1s.

Next, the expression of HuR was compared in KM12C and KM12L4 cells. Interestingly, the western blot results revealed that the expression level of HuR in both cells was almost similar (Fig. 7D). Moreover, the cellular localization of HuR did not differ between cells (Fig. 7E). We investigated the effect of the circPPFIA1s on HuR expression and vice versa. When circPPFIA1-L and -S were silenced in KM12C cells, the expression level and cellular localization of HuR were unchanged (Fig. 7 F,G). Moreover, the knockdown of HuR by two independent siRNAs did not cause notably altered expression levels of circPPFIA1-L and -S in KM12L4 cells (Fig. 7H). Based on these results, we assumed that circPPFIA1s may affect the regulatory functions of HuR, such as stabilization or translational activation of its target mRNA, without any change in the expression and localization of HuR.

To test whether HuR can regulate metastatic potential, the invasive and migratory abilities of KM12L4 cells were examined by transwell assays. The knockdown of HuR dramatically decreased the number of invaded and migrated cells (Fig. 7I). We also determined whether HuR is required for the increased metastatic potential of KM12C cells by lowering the expression of circPPFIA1s. Increased metastatic potential by the knockdown of circPPFIA1s was reversed through HuR silencing (Fig. 7J). This indicated that HuR is required for the increase in metastatic ability due to the knockdown of circPPFIA1s. As expected, when the expression level of HuR was lowered, the metastatic potential was decreased, and when circPPFIA1s were silenced, KM12C cells showed high metastatic potential. However, the knockdown of both HuR and circPPFIA1s decreased the invaded and migrated cell number compared to those of circPPFIA1-silenced cells.

### RAB36 is involved in the control of metastatic potential by circPPFIA1s/HuR

**Fig. 8:**
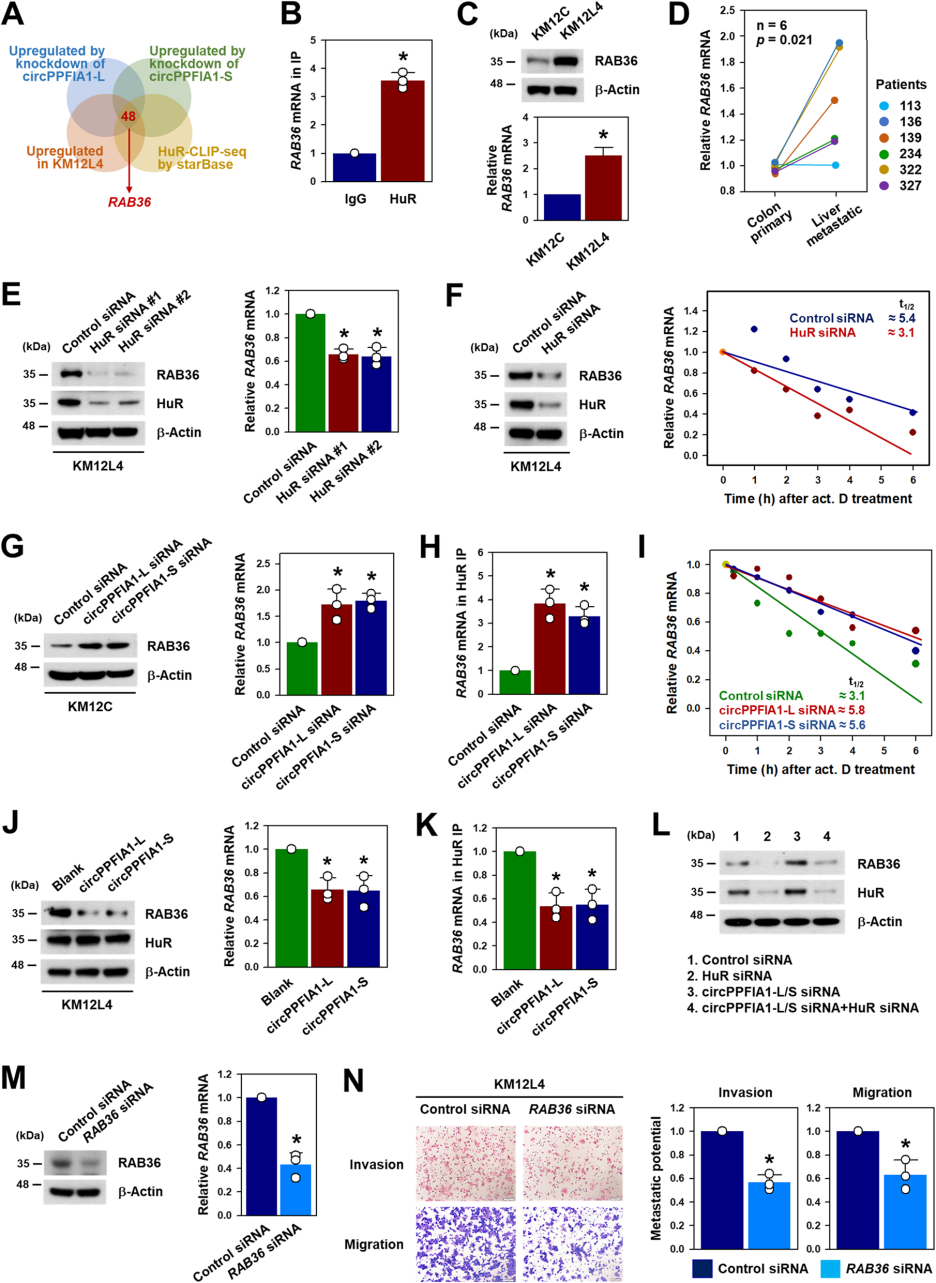
CircPPFIA1 negatively regulates RAB36 expression by sequestering HuR. **A** Venn diagram representing overlapping genes by comparing RNA sequencing data with HuR CLIP-seq data. **B** Association of HuR with *RAB36* mRNA was checked by HuR RIP. The enrichment of *RAB36* mRNA in HuR RIP was determined by RT-qPCR. **C** The expression levels of RAB36 protein and mRNA were compared in KM12C and KM12L4 cells by western blot and RT-qPCR analyses, respectively. **D** The expression level of *RAB36* in primary and liver metastatic colon cancer tissues was determined by RT-qPCR. **E** Western blot and RT-qPCR analyses were used to detect RAB36 protein and mRNA upon HuR silencing by two independent siRNAs. **F** The effect of HuR silencing on the stability of *RAB36* mRNA was examined in KM12L4 treated with actinomycin D at the indicated time point. Half-lives of RAB36 mRNA were determined by calculating the time (t_1/2_) with 50% mRNA remaining. **G**–**I** The effect of circPPFIA1s silencing on RAB36 expression was examined by western blot analysis (**G**), HuR RIP (**H**), and the stability assay (**I**) using actinomycin as described above. **J**, **K** To examine whether overexpression of circPPFIA1s decreases RAB36 expression, KM12L4 cells were transfected with blank or overexpression vectors. The expression levels of RAB36 protein and mRNA were determined by western blot and RT-qPCR, respectively (**J**). The effect of circPPFIA1s overexpression on the interaction between HuR and *RAB36* mRNA was examined by HuR RIP (**K**). **L **To examine whether knockdown of HuR reverses upregulation of RAB36 by knockdown of circPPFIA1s, western blot analysis was performed. **M**, **N** Following the transfection of KM12L4 cells with RAB36 siRNA, the efficacy of CDX1 siRNA was examined by western blot and RT-PCR (**M**). The effect of RAB36 silencing on the metastatic potential of KM12L4 cells was checked by transwell invasion and migration assays (**N**). Statistical analyses were performed using the Student’s *t*-test using three independent experiments (*p < 0.05). All data represent mean ± standard variation (SD)

By comparing and analyzing the HuR CLIP-sequencing results with the list of genes upregulated under the three described conditions, 48 out of 62 genes (approximately 77% of the total merged genes) were found to be putative HuR target genes (Supplementary Figure S21). Among these genes, *RAB36* was selected as a HuR target gene using the reference investigation (Fig. 8A). To verify that *RAB36* is a HuR target, an HuR RIP experiment was conducted. The level of *RAB36* mRNA was more enriched in HuR IP compared to IgG IP (Fig. 8B). Western blot and RT-qPCR analyses indicated that the expression levels of RAB36 protein and mRNA were higher in KM12L4 cells (Fig. 8C). Similar to what was observed in cells, *RAB36* was highly expressed in liver metastatic colon cancer tissues compared to primary colon cancer tissues (Fig. 8D). Moreover, the knockdown of HuR by two independent siRNAs downregulated RAB36 protein and mRNA, indicating that *RAB36* is a novel target of HuR (Fig. 8E).

As an oncogene, the main mechanism of HuR is the stabilization of target mRNA by directly binding to its 3’UTR, which results in the upregulation of the target gene. Therefore, we determined whether HuR increased the stability of *RAB36* mRNA. The decreased expression of RAB36 by knockdown of HuR was confirmed using the mixture of HuR siRNAs (Fig. 8F). The knockdown of HuR induced a more rapid decrease in *RAB36* mRNA compared to that in the control (Fig. 8F). The estimated half-lives of *RAB36* mRNA in the control and HuR-silenced KM12L4 cells were 5.4 h and 3.1 h, respectively. Next, we investigated the functional role of circPPFIA1s in HuR-mediated RAB36 regulation. The knockdown of circPPFIA1-L and -S increased the expression level of RAB36 protein and mRNA (Fig. 8G). The HuR RIP experiment indicated that the knockdown of circPPFIA1s enhanced the interaction between HuR and *RAB36* mRNA, which allowed HuR to stabilize *RAB36* mRNA (Fig. 8H). Although the estimated half-lives of *RAB36* mRNA in the control was approximately 3.1 h, it increased to 5.8 h and 5.6 due to the knockdown of circPPFIA1-L and -S, respectively (Fig. 8I). Conversely, the overexpression of circPPFIA1-L and -S induced a decrease in RAB36 protein and mRNA (Fig. 8J) and lowered the enrichment of RAB36 mRNA in HuR IP materials (Fig. 8K).

We assessed the expression level of RAB36 in the same samples, because *RAB36* was identified as a HuR target. Increased level of RAB36 by the knockdown of circPPFIA1s was lowered by HuR silencing (Fig. 8L). This indicates that the liberation of HuR by decreasing the levels of circPPFIA1s is required for highly metastatic phenotypes. We also tested whether RAB36 is involved in the invasive and migratory abilities of KM12L4 cells. Introducing a siRNA that targets *RAB36* mRNA efficiently decreased the expression of RAB36 protein and mRNA in KM12L4 cells (Fig. 8M). The metastatic potential was also diminished by the knockdown of RAB36 (Fig. 8N).

**Fig. 9:**
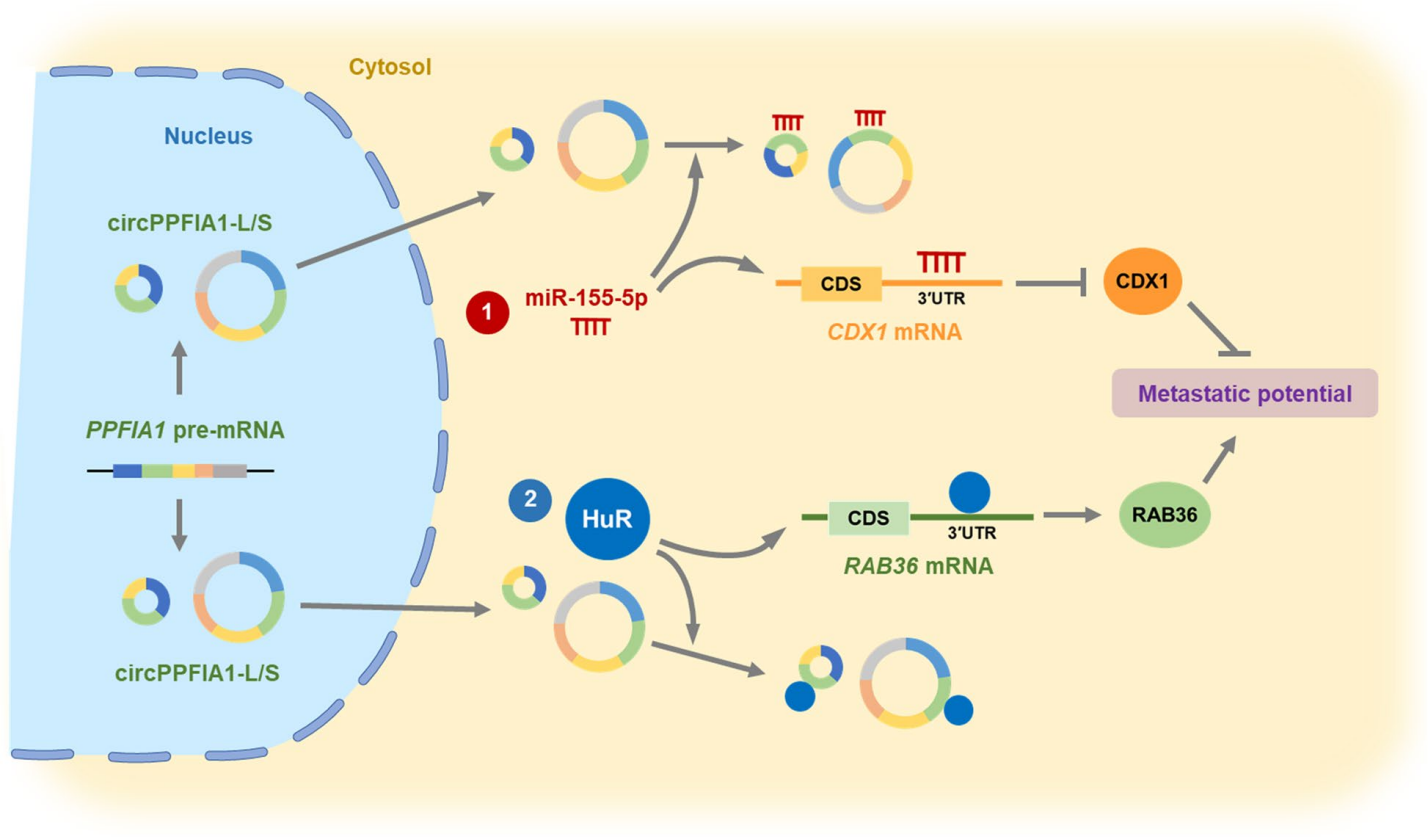
Summarized schematic illustration showing the inhibitory effect of circPPFIA1s on the metastatic potential of CRC

Our findings are summarized by a schematic illustration in Fig. 9. Briefly, two circPPFIA1s, generated from the exons of the *PPFIA1* gene, are downregulated in the liver metastasis of CRC. They are mainly present in the cytosol, which allows them to function as molecular sponges. As tumor suppressors, circPPFIA1-L and -S negatively control the metastatic potential of CRC via two pathways: as a sponge of miR-155-5p, upregulating CDX1 expression; and as a sponge of HuR, downregulating RAB36 expression.

## Discussion

Emerging evidences indicate that circRNAs are closely associated with various diseases, especially with cancers. Their unique nature and specificity made them a new hotspot in the field of biomedical research in recent years. In this study, we identified two circRNAs, circPPFIA1-L and -S, downregulated in liver metastatic KM12L4 cells compared to primary KM12C cells through circRNA microarray. Functionally, circPPFIA1s carry anti-metastatic roles in CRC by sponging oncogenic miR-155-5p and HuR. Thus, our finding suggests that two circPPFIA1s may be used for potential clinical diagnosis of CRC.

CircRNAs play a critical role in the progression of CRC. According to the circRNA profile of CRC, approximately 75–80% of differentially expressed circRNAs (DECs) are derived from exons [22, 23]. Most exon-containing circRNAs are predominantly located in the cytosol, generally functioning as molecular sponges [24]. Tumor-suppressive circRNAs (for example circ_001988, circ_0009361, and circ_0021977), like other tumor suppressors, are downregulated in CRC, which enhances the inhibitory effect of oncogenic miRNAs and accordingly suppresses the tumor-suppressing target genes [25–27]. Here, we found that two circPPFIA1s (circPPFIA1-L and -S), generated from the exons of *PPFIA1*, are downregulated in liver metastatic colon cancer cells and tissues.

Liprin-α1, encoded by *PPFIA1*, interacts with the leukocyte common antigen-related family of tyrosine phosphatases and plays an important role in axon guidance and mammary gland development [28]. In addition to its function in neuronal cells, liprin-α1 is associated with the proliferation, migration, and invasion of cancer cells [29, 30]. According to the circBase database (www.circbase.org), 37 circRNAs are generated from *PPFIA1* (Supplementary Figure S3). Here, we identified two anti-metastatic circRNAs generated from *PPFIA1*. There are several reports on the function of *PPFIA1*-generated circRNAs in cancer. CircPPFIA1 enhances the metastatic potential of laryngeal squamous cell carcinoma via miR-340-3p/ELK1 [31]. Despite shared nomenclature, they are different circRNAs. CircPPFIA1 is circ_0023326, but the circPPFIA1-L and -S reported here are circ_0003426 and circ_0000337, respectively. circPPFIA1-L and -S, previously reported under the names circRNA_100873 [32] and circ_0000337 [33], are associated with the lymphatic metastasis of esophageal squamous cell carcinoma. However, its detailed action mechanism is not fully understood in CRC.

Accumulating evidences shown that circRNAs exerted diverse biological functions by serving as sponges for miRNAs. As competing endogenous RNA (ceRNA), circRNAs regulate miRNA function by inhibiting binding of miRNAs to 3’UTR of their targets. For example, circHIPK3 promotes CRC progression and metastasis by sponging miR-7 [34]. CircSAPRC enhances metastatic potential of CRC through miR-485-3p/JAK2 [35]. Herein, we found that circPPFIA1s inhibit CRC metastasis by mitigating the inhibitory function of miR-155-5p. MiR-155-5p, a well-known oncogenic miRNA, promotes oral cancer progression by suppressing the chromatin remodeling gene *ARID2* [36]. Additionally, miR-155-5p is associated with the anti-tumor effect of cetuximab, cisplatin, and 5-FU in breast cancer, hepatocellular carcinoma cells (HCCs), and CRC, respectively [37–39]. In our study, based on analysis of RNA-seq data and bioinformatic tools, *CDX1* is identified as a target of circPPFIA1s/miR-155-5p. *CDX-1*, an intestine-specific gene, is generally downregulated in CRC [40, 41] and acts as a tumor-suppressor by hindering the transcriptional activity of β-catenin/T-cell factor [42]. *CDX1* induction also alters the transcript expression of genes related to cell adhesions for EMT and angiogenesis [40]. As miR-155-5p sponge, circPPFIA1s increase the expression of CDX1 by blocking the interaction of mIR-155-5p with *CDX1* mRNA, resulting in lowered metastatic potential of CRC.

There has been controversy over whether circRNA can regulate the expression of miRNA. However, emerging evidences have revealed that circRNA as a ceRNA could not affect the level of miRNA since they do not degrade their sponging miRNA. CircTLK1 did not influence the expression level of miR-136-5p but inhibited its inhibitory effect on CBX4 expression as a miRNA sponge [43]. Moreover, a well-known circRNA, CDR1as which contains 63 conserved MREs for miR-7 suppressed its activity without affecting the expression of miR-7 [44]. CircFOXK2 was found to hinder the function of miR-942 without the alteration of its expression level [45]. The circular RNA circRIP2 was also reported to regulate the suppressing effect of miR-1305 but not its expression [46]. Our data demonstrate that circPPFIA1s act as tumor suppressors by sponging the oncogenic miR-155-5p.

In addition to miRNA sponge, circRNAs also affect the function of RBP as a RBP sponge. For example, circPTPRA suppresses bladder cancer progression by blocking the interaction between IGF2BP1 and its target mRNAs [47]. For exploring the additional function of circPPFIA1s on RBP, we searched for circPPFIA1s-associated RBP with the bioinformatic tools and found that ELAV-like protein HuR is a binding partner of circPPFIA1s. HuR is closely related to malignant phenotypes of CRC mainly through the stabilization of its target mRNA [48]. Our findings demonstrated that increased metastatic potential by knockdown of circPPFIA1s was attenuated through HuR silencing, thus hypothesizing that circPPFIA1s is responsible for blocking oncogenic effects of HuR in CRC. Several tumor-suppressing circRNAs function as HuR sponges. CircRHOBTB3 suppresses CRC metastasis by hindering the HuR-mediated stabilization of polypyrimidine tract-binding protein 1 (*PTBP1)* mRNA [49]. CircDLC1, a prognostic marker of hepatocellular carcinoma (HCC), inhibits the motility of HCC by sequestering HuR from matrix metalloproteinase-1 (*MMP1)* mRNA [50]. By a similar action mechanism, circPPFIA1s can influence HuR targets by sponging it. We found that RAB36 is the downstream effector molecule of circPPFIA1s. RAB36, a member RAS oncogene family, is upregulated in various types of cancers and may be closely related with tumor development and metastasis. In bladder cancer, it promotes cancer progression and invasion [51]. Accordingly, RAB36 is a target of circPPFIA1s/HuR.

## Conclusion

We searched for metastasis-associated circRNAs using KM12C CRC cells and its liver metastatic derivative KM12L4. Using a circRNA microarray, we identified several circRNAs downregulated in KM12L4 cells compared to KM12C cells. Two circPPFIA1s, generated from *PPFIA1*, were significantly downregulated in liver metastatic cells. The circular structure of circPPFIA1s was verified by RNase R resistance and Sanger sequencing. Using in vitro transwell assays and in vivo intrasplenic injection mouse experiments, we found that circPPFIA1s negatively regulated the liver metastasis of CRC. Mechanistically, an ASO pulldown assay revealed that both circPPFIA1-L and -S function as sponges for oncogenic miR-155-5p and HuR. circPPFIA1s upregulate tumor-suppressing CDX1 by decoying *CDX1*-targeting miR-155-5p and downregulate oncogenic RAB36 by sequestering HuR. Taken together, circPPFIA1s, as a metastasis suppressor, are a promising therapeutic target for the treatment of the liver metastasis of CRC.

## Supplementary Information

**Additional file 1:** **Supplementary Table S1.** Verification of cell lines used in this study by STR analysis. **Supplementary Table S2.** PCR primers and siRNAs used in this study. **Supplementary Table S3.** List of antibodies used in this study. **Supplementary Table S4.** Composition of buffers used in this study. **Supplementary methods**. **Supplementary Figure S1.** Comparison of metastatic potential of primary (KM12C) and liver metastatic colorectal cancer cells (KM12L4). **A** Schematic illustration of the establishment of the liver metastatic colorectal cancer cell model. KM12C was originally obtained from a human specimen and KM12L4 was generated through the fourth selection-isolation of intrasplenic injection. **B** Liver metastases were examined using in vivo intra-splenic injection. At four weeks post-injection, optical and MRI images were obtained. The degree of liver metastasis (*n* = 5) was calculated by giving scores in arbitrary units (0–3). **Supplementary Figure S2. **Analyses of circRNA microarray data. **A** A heat map analysis of circRNA microarray. **B** A volcano plot of circRNA microarray data showing a list of the upregulated and downregulated circRNAs. **C** Detailed information on circPPFIA1-L and -S. **Supplementary Figure S3.** List of *PPFIA1*-originated circRNAs. circRNAs that are generated from the *PPFIA1 *gene are listed in a public circRNA database (circBase, http://www.circbase.org). **Supplementary Figure S4.** Primer sequences used in this study. **A** Schematic illustration and sequences of RT-qPCR primers detecting circPPFIA1-L, circPPFIA1-S, and linear *PPFIA1*. **B** Validation of the expression of circPPFIA1-L and -S by RT-qPCR using the above primer sets. **C** Schematic illustration and sequences of semi-qPCR primers detecting circPPFIA1-L, circPPFIA1-S, and linear *PPFIA1*. **D** Schematic illustration and sequences of primers for Sanger sequencing. **Supplementary Figure S5.** Comparison of circPPFIA1-L and -S expression between adjacent normal and tumor tissues of CRC patients. Tumor tissues and their matched normal tissues were obtained from 14 CRC patients at the Samsung Medical Center. Among the 14 patients, seven did not exhibit metastasis while the other seven patients showed liver metastasis. All samples were collected with the informed consent of patients under institutional review board-approved protocols and stored at -80°C until use. The expression level of circPPFIA1-L and -S was determined by RT-qPCR (*n* = 38 for circPPFIA1-L, *n* = 28 for circPPFIA1-S). **Supplementary Figure S6.** Characterization of circPPFIA1-L and -S. **A** The stability of circPPFIA1s and linear *PPFIA1 *was examined by RT-qPCR using total RNA isolated from actinomycin D-treated KM12C cells. **B** Schematic illustration of divergent and convergent semi-qPCR primers for circPPFIA1-L and -S. Semi-qPCR analysis was conducted using genomic (gDNA) and complementary (cDNA) DNA. *GAPDH *was used as a negative control. **C** Sanger sequencing results of circPPFIA1-L and -S. **Supplementary Figure S7.** Design and validation of siRNAs targeting circPPFIA1-L and -S. (A, C) Schematic illustration and sequences of circPPFIA1-L siRNAs (A), and -S (C). (**B**, **D**) Each siRNA efficiently decreases the expression level of circPPFIA1-L (B) and -S (D). (**E**) For a cotransfection experiment, KM12C cells were simultaneously transfected with siRNAs targeting circPPFIA1-L and -S. The expression levels of circPPFIA1s and *GAPDH *were determined by semi-qPCR. **Supplementary Figure S8.** Potentiation of metastatic properties by knockdown of circPPFIA1-L and -S in KM12C cells. (**A**, **B**) KM12C cells were transfected with the indicated siRNA (shown in Supplementary Figure S7), and transwell assays were performed to determine invasive (**A**) and migratory (**B**) abilities. (C, **D**) Cell proliferation of KM12C cells transfected with individual or a mixture of siRNAs targeting circPPFIA1-L (**C**) or -S (**D**) was determined by counting the number of viable cells. **Supplementary Figure S9.** Increase in liver metastasis in vivo by knockdown of circPPFIA1s. The effect of circPPFIA1-L and -S on liver metastasis in vivo was examined through intrasplenic injection of KM12C cellstransfected with circPPFIA1s siRNAs. (**A**, **C**) The expression level of circPPFIA1-L (**A**) and -S (**C**) was determined by semi-qPCR. (**B**, **D**) Liver metastases were examined using in vivo intrasplenic injection. At four weeks post-injection, optical and MRI images were obtained. The degree of liver metastasis (*n* = 7) was calculated by giving scores in arbitrary units (0–3). **Supplementary Figure S10.** Suppression of metastatic potential by overexpression of circPPFIA1-L and -S in KM12L4 cells. (**A**-**C**) By introducing the circPPFIA1-L overexpression vector into KM12L4 cells, three independent clones (#1–#3) were generated and were used to investigate the effect of circPPFIA1-L on metastatic potential. Invasive ability was examined by transwell invasion assays, and the expression levels of circPPFIA1-L and *PPFIA1 *mRNA were measured by RT-qPCR. (**D**) In the same way as above, two clones (#1 and #2) in which circPPFIA1-S was overexpressed were generated, and invasive and migratory abilities were determined by transwell invasion and migration assays. (**E**) The effect of circPPFIA1 overexpression on cell proliferation was examined by counting the number of viable cells. **Supplementary Figure S11.** Decrease in liver metastasis in vivo by overexpression of circPPFIA1s. The effect of circPPFIA1-L and -S on liver metastasis in vivo was examined via intrasplenic injection of KM12L4 cells, wherein circPPFIA1-L or -S was overexpressed. (**A, C**) The expression levels of circPPFIA1-L (**A**) and -S (**C**) were determined by semi-qPCR. (**B**, **D**) Liver metastases were examined via in vivo intrasplenic injection of circPPFIA1-L (**B**) or -S (**D**) overexpressing KM12L4 cells. At four weeks post-injection, optical and MRI images were obtained. The degree of liver metastasis (*n* = 7) was calculated by giving scores in arbitrary units (0–3). **Supplementary Figure S12.** Negative regulation of invasive and migratory abilities via circPPFIA1-L and -S in DLD1 and RKO cells. (**A**, **B**) Following transfection of DLD1 (**A**) and RKO (**B**) cells with indicated siRNA, transwell assays were conducted to measure invasive and migratory abilities. The expression levels of circPPFIA1-L, -S, and *PPFIA1 *mRNA were determined by semi-qPCR. *GAPDH *was used as a loading control. (**C**, **D**) For overexpression of circPPFIA1-L and -S, DLD1 (**C**) and RKO (**D**) cells were transfected with indicated vector. The number of invaded and migrated cells was assessed using transwell assays. The expression levels of circPPFIA1-L, -S, and *PPFIA1 *mRNA were determined by semi-qPCR. *GAPDH *was used as a loading control. **Supplementary Figure S13.** Venn diagram for selecting putative interacting miRNAs with circPPFIA1. By prediction of circPPFIA1-interacting miRNAs using four prediction algorithms (ArrayStar, RNA22, circInteractome, and Starbase), miR-155-5p was selected for further studies. **Supplementary Figure S14.** Schematic illustration and design of antisense oligonucleotide (ASO) for the pulldown experiments. (**A**, **B**) ASOs for pulldown were designed to bind to the divergent region of circPPFIA1-L (A) or -S (**B**). circPPFIA1-L and -S were captured using three and two ASOs, respectively. The enrichment of the corresponding circRNA in pulldown materials was assessed by semi-qPCR. (**C**) The sequences of ASOs for pulldown experiments. *LacZ *was used as a control ASO. **Supplementary Figure S15.** Construction of luciferase vectors harboring wild-type (WT) or mutant (MT) sequences of miR-155-5p miRNA recognition element (MRE) in circPPFIA1-L and -S. (**A**) Two miR-155-5p MREs (#1 and #2) were predicted by bioinformatics approaches in the sequence of exon 18 of *PPFIA1*. (**B**) Dual-luciferase vectors harboring wild-type (WT) or mutant (MT) sequences of each miR-155-5p MRE were manufactured. **Supplementary Figure S16.** Regulation of metastatic potential by miR-155-5p in DLD1 and RKO cells. DLD1 (**A**) and RKO (**B**) cells were transfected with pre-miR-155-5p or anti-miR-155-5p. Invasive and migratory abilities were assessed using transwell invasion and migration assays. **Supplementary Figure S17.** Rescue experiment for proving that miR-155-5p is required for the increase in metastatic potential by knockdown of circPPFIA1. KM12C cells were simultaneously transfected with circPPFIA1 siRNA and anti-miR-155-5p. Invasive and migratory abilities were examined using transwell invasion (**A**) and migration (**B**) assays. **Supplementary Figure S18.** Venn diagram for screening common target genes that are regulated via circPPIFIA1s/miR-155-5p. By comparing the lists of downregulated genes obtained from RNA sequencing data, and miR-155-5p target genes predicted by miRDB and TargetScan, six genes (ETS1, CDX1, TCF4, TP53INP1, MAFB, and BDNF) were identified as putative targets. **Supplementary Figure S19.** Schematic of dual-luciferase reporter vectors harboring wild-type or mutant sequences of miR-155-5p MRE in *CDX1 *mRNA.** Supplementary Figure S20.** Prediction and validation of circPPFIA1-interacting RNA-binding proteins. (**A**) Venn diagram for selecting circPPFIA1-interacting RBPs using three prediction algorithms (circInteractome, Starbase, and RBPDB). (**B**) The levels of predicted RBPS in ASO pulldown materials were assessed by western blot analyses using the indicated antibodies. (**C**) ASO pulldown followed by western blot analysis was conducted to verify the interaction of HuR with circPPFIA1s (left, circPPFIA1-L; right, circPPFIA1-S). **Supplementary Figure S21.** High proportion of common target genes harbor the HuR-binding motif.Upregulated common target genes were screened based on the following three criteria: (i) genes upregulated genes by knockdown of circPPFIA1-L, (ii) genes upregulated genes by knockdown of circPPFIA1-S, (iii) genes upregulated in KM12L4 compared to KM12C. By comparing and analyzing the public data of HuR CLIP-seq with the selected common target genes, we observed that 77% (48 genes out of 62 genes) of selected target genes have HuR-binding motifs in their 3’-UTRs.

## Data Availability

All data and materials analyzed or used to support the findings in this study are available from the corresponding author upon a reasonable request.

## Abbreviations

CRC: Colorectal cancer
circRNA: Circular RNA
miRNA: MicroRNA
RBP: RNA-binding protein
PPFIA1: PTPRF-interacting protein alpha 1
ceRNA: Competitive endogenous RNA
CDX1: Caudal type homeobox 1
HuR: Hu antigen R
RAB36: Member RAS oncogene family 36
RIP: Ribonucleoprotein immunoprecipitation
AGO2: Argonaute 2
siRNA: Small interfering RNA
3’UTR: 3’-untranslated region
ASO: Antisense oligonucleotide
