# Supplementary material for: Two circPPFIA1s negatively regulate liver metastasis of colon cancer via miR-155-5p/CDX1 and HuR/RAB36
Source: Mol Cancer. 2022 Oct 12;21:197. doi: 10.1186/s12943-022-01667-w (PMC9555114; doi:10.1186/s12943-022-01667-w)
Supplement: Supplementary file 1 — Additional file 1: Supplementary Table S1. Verification of cell lines used in this study by STR analysis. Supplementary Table S2. PCR primers and siRNAs used in this study. Supplementary Table S3. List of antibodies used in this study. Supplementary Table S4. Composition of buffers used in this study. Supplementary methods. Supplementary Figure S1. Comparison of metastatic potential of primary (KM12C) and liver metastatic colorectal cancer cells (KM12L4). A Schematic illustration of the establishment of the liver metastatic colorectal cancer cell model. KM12C was originally obtained from a human specimen and KM12L4 was generated through the fourth selection-isolation of intrasplenic injection. B Liver metastases were examined using in vivo intra-splenic injection. At four weeks post-injection, optical and MRI images were obtained. The degree of liver metastasis (n = 5) was calculated by giving scores in arbitrary units (0–3). Supplementary Figure S2. Analyses of circRNA microarray data. A A heat map analysis of circRNA microarray. B A volcano plot of circRNA microarray data showing a list of the upregulated and downregulated circRNAs. C Detailed information on circPPFIA1-L and -S. Supplementary Figure S3. List of PPFIA1-originated circRNAs. circRNAs that are generated from the PPFIA1 gene are listed in a public circRNA database (circBase, http://www.circbase.org). Supplementary Figure S4. Primer sequences used in this study. A Schematic illustration and sequences of RT-qPCR primers detecting circPPFIA1-L, circPPFIA1-S, and linear PPFIA1. B Validation of the expression of circPPFIA1-L and -S by RT-qPCR using the above primer sets. C Schematic illustration and sequences of semi-qPCR primers detecting circPPFIA1-L, circPPFIA1-S, and linear PPFIA1. D Schematic illustration and sequences of primers for Sanger sequencing. Supplementary Figure S5. Comparison of circPPFIA1-L and -S expression between adjacent normal and tumor tissues of CRC patients. Tumor tissues and their [file 12943_2022_1667_MOESM1_ESM.pdf]

A

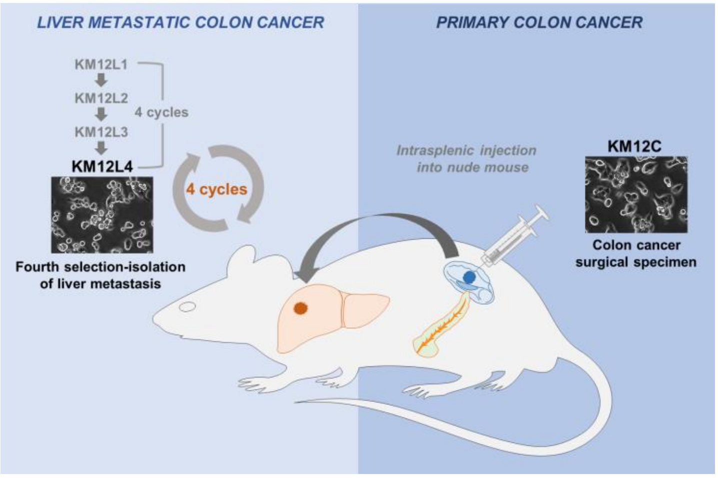

B

	1	2	3	4	5
**KM12C** *Incidence=40% (2/5)*	-	++	+	+	-
**KM12L4** *Incidence=100% (5/5)*	+++	+++	+	++	+++

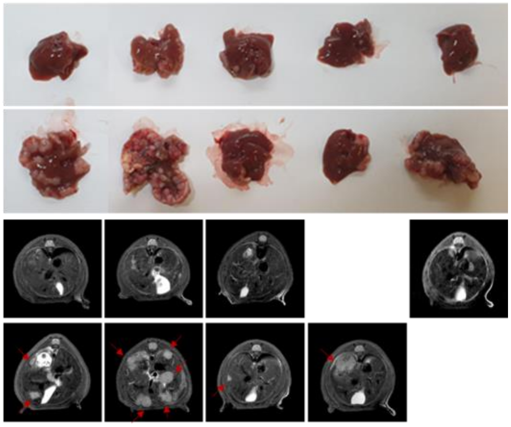

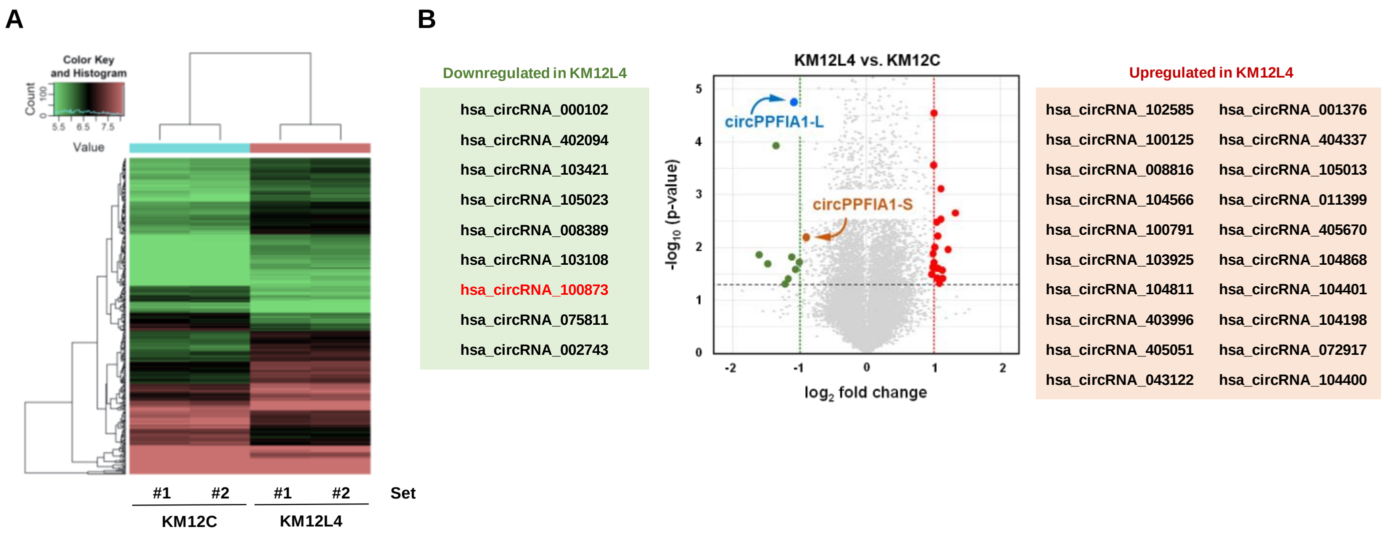

C

	KM12L4 vs. KM12C		circRNA ID		Location	Length	
	Fold	*p* value				Genomic	Spliced
circPPFIA1-L	-2.13	0.00002	circRNA_100873	hsa_circ_0003429	chr11: 70200406-70208594	8188 bp	702 bp
circPPFIA1-S	-1.91	0.01126	circRNA_100872	hsa_circ_0000337	chr11: 70200406-70202360	1954 bp	419 bp

circRNA ID	Position	Strand	Genomic length	Spliced length	Best transcript	Gene symbol
**hsa_circ_0000337**	**chr11:70200406-70202360**	**+**	**1954**	**419**	**NM_177423**	**PPFIA1**
hsa_circ_0002767	chr11:70196028-70202360	+	6332	1520	NR_045286	PPFIA1
**hsa_circ_0003429**	**chr11:70200406-70208594**	**+**	**8188**	**702**	**NR_045286**	**PPFIA1**
hsa_circ_0006538	chr11:70218320-70224301	+	5981	685	NR_045286	PPFIA1
hsa_circ_0023313	chr11:70118278-70176425	+	58147	1077	NR_045286	PPFIA1
hsa_circ_0023314	chr11:70118278-70194526	+	76248	2163	NM_003626	PPFIA1
hsa_circ_0023315	chr11:70118278-70208594	+	90316	2876	NR_045286	PPFIA1
hsa_circ_0023316	chr11:70170507-70194526	+	24019	1899	NM_003626	PPFIA1
hsa_circ_0023317	chr11:70171605-70208594	+	36989	2345	NR_045286	PPFIA1
hsa_circ_0023318	chr11:70172367-70194526	+	22159	1557	NM_003626	PPFIA1
hsa_circ_0023319	chr11:70172367-70200558	+	28191	1720	NR_045286	PPFIA1
hsa_circ_0023320	chr11:70172702-70194526	+	21824	1455	NM_003626	PPFIA1
hsa_circ_0023321	chr11:70176278-70194526	+	18248	1233	NM_003626	PPFIA1
hsa_circ_0023322	chr11:70178065-70200558	+	22493	1249	NR_045286	PPFIA1
hsa_circ_0023323	chr11:70183470-70194526	+	11056	735	NM_003626	PPFIA1
hsa_circ_0023324	chr11:70185276-70194526	+	9250	592	NR_045286	PPFIA1
hsa_circ_0023325	chr11:70189774-70194526	+	4752	456	NR_045286	PPFIA1
hsa_circ_0023326	chr11:70194294-70202360	+	8066	681	NR_045286	PPFIA1
hsa_circ_0023327	chr11:70197099-70222706	+	25607	1251	NR_045286	PPFIA1
hsa_circ_0023328	chr11:70200406-70218496	+	18090	878	NR_045286	PPFIA1
hsa_circ_0023329	chr11:70200406-70221199	+	20793	1152	NR_045286	PPFIA1
hsa_circ_0023330	chr11:70200406-70222706	+	22300	1221	NR_045286	PPFIA1
hsa_circ_0023331	chr11:70200406-70224301	+	23895	1387	NR_045286	PPFIA1
hsa_circ_0023332	chr11:70202266-70202360	+	94	94	NR_045286	PPFIA1
hsa_circ_0023333	chr11:70222637-70224301	+	1664	235	NR_045286	PPFIA1
hsa_circ_0096341	chr11:70170507-70172924	+	2417	666	NR_045286	PPFIA1
hsa_circ_0096342	chr11:70170507-70178200	+	7693	948	NR_045286	PPFIA1
hsa_circ_0096343	chr11:70176278-70178200	+	1922	282	NR_045286	PPFIA1
hsa_circ_0096344	chr11:70176278-70181800	+	5522	498	NR_045286	PPFIA1
hsa_circ_0096345	chr11:70178065-70181800	+	3735	351	NR_045286	PPFIA1
hsa_circ_0096346	chr11:70189774-70200558	+	10784	638	NR_045286	PPFIA1
hsa_circ_0096347	chr11:70189774-70202360	+	12586	905	NR_045286	PPFIA1
hsa_circ_0096348	chr11:70197099-70202360	+	5261	449	NR_045286	PPFIA1
hsa_circ_0096350	chr11:70208200-70222706	+	14506	802	NR_045286	PPFIA1
hsa_circ_0096352	chr11:70218317-70222706	+	4389	522	NR_045286	PPFIA1
hsa_circ_0096353	chr11:70221023-70222706	+	1683	245	NR_045286	PPFIA1
hsa_circ_0140970	chr11:70221023-70222706	+	1683	245	NR_045286	PPFIA1

A

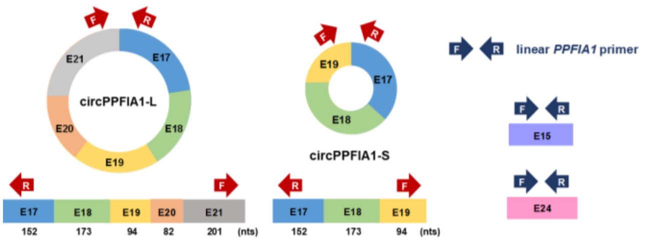

circPPFIA1L	RT-qPCR primer#1	Forward	CGCCCACATCTAGAACGTT
		Reverse	CGGGGAGGTTTCACACTTTA
	RT-qPCR primer#2	Forward	CCACATCTAGAACGTTGCCA
		Reverse	GAAGGCGGGGAGGTTTCA
	RT-qPCR primer#3	Forward	AGGAGATCATGTCGCTGACC
		Reverse	GGTTGTCTTGTCATCTCGTACC
circPPFIA1S	RT-qPCR primer#1	Forward	AGGATGCCTTGGGACTTAGC
		Reverse	TCTCTGGAAGGTGGCAATTTT
	RT-qPCR primer#2	Forward	CTTCAAAAAAATTGCCACCTTCC
		Reverse	GGTCTAACCGAAGGGCTCT
PPFIA1	RT-qPCR primer#1	Forward	CGAGAGTGATGCTGACGTGT
		Reverse	GCATCATGGCTAGTGTGTGC
	RT-qPCR primer#2	Forward	TGGATCCTGTCAATTGGCCT
		Reverse	TCTAAGGCCAGAAGTGCTCC

B

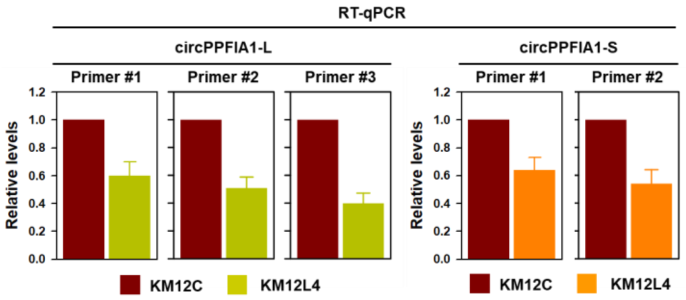

C

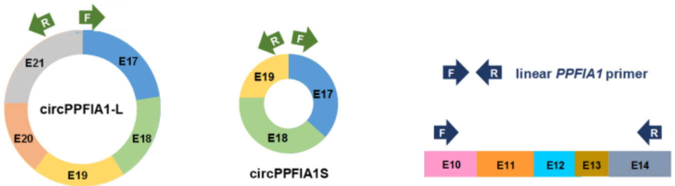

circPPFIA1L	Semi-qPCR primer	Forward	CCCACATCTAGAACGTTGCC
		Reverse	GGCTGGTCAGCGACATGAT
circPPFIA1S	Semi-qPCR primer	Forward	TTCAAAAAAATTGCCACCTTCC
		Reverse	CGATTTTTTTCAGCCTGTCCC
PPFIA1	Semi-qPCR primer	Forward	ATGGAAGCACAGTTGGAGGA
		Reverse	GGTGCTGTAGGAGTCTGTGT

D

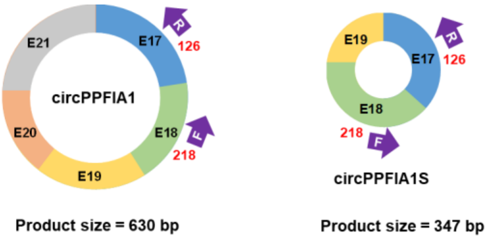

circPPFIA1	Sanger primer	Forward	CCACAAAGCCCCAAAGAAGA
		Reverse	ATGTCCCTGATGTCCTCGTG

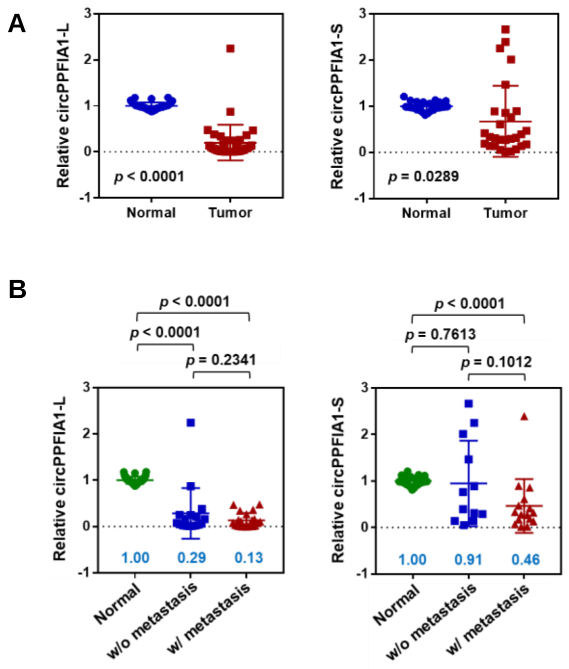
A
Relative circPPFIA1-L
p < 0.0001
Normal
Tumor
Relative circPPFIA1-S
p = 0.0289
Normal
Tumor
B
p < 0.0001
p < 0.0001
p = 0.2341
Relative circPPFIA1-L
1.00
0.29
0.13
Normal
w/o metastasis
w/ metastasis
p < 0.0001
p = 0.7613
p = 0.1012
Relative circPPFIA1-S
1.00
0.91
0.46
Normal
w/o metastasis
w/ metastasis

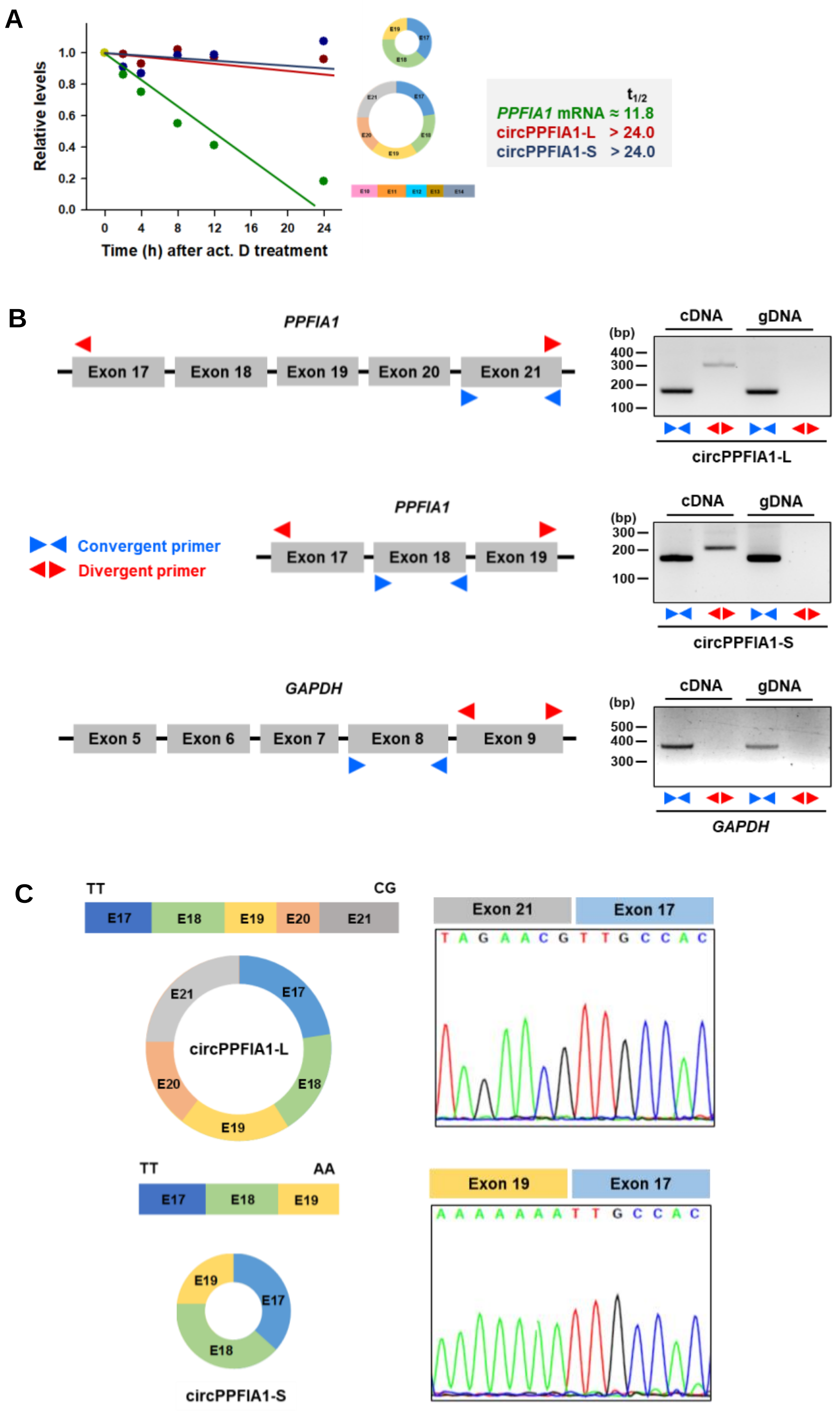
A
Relative levels
1.0
0.8
0.6
0.4
0.2
0.0
0
4
8
12
16
20
24
Time (h) after act. D treatment
t1/2
PPFIA1 mRNA ≈ 11.8
circPPFIA1-L > 24.0
circPPFIA1-S > 24.0
B
PPFIA1
Exon 17
Exon 18
Exon 19
Exon 20
Exon 21
cDNA
gDNA
(bp)
400
300
200
100
circPPFIA1-L
Convergent primer
Divergent primer
PPFIA1
Exon 17
Exon 18
Exon 19
(bp)
300
200
100
circPPFIA1-S
GAPDH
Exon 5
Exon 6
Exon 7
Exon 8
Exon 9
(bp)
500
400
300
GAPDH
C
TT
CG
E17
E18
E19
E20
E21
circPPFIA1-L
Exon 21
Exon 17
T A G A A C G T T G C C A C
TT
AA
E17
E18
E19
circPPFIA1-S
Exon 19
Exon 17
A A A A A A A T T G C C A C

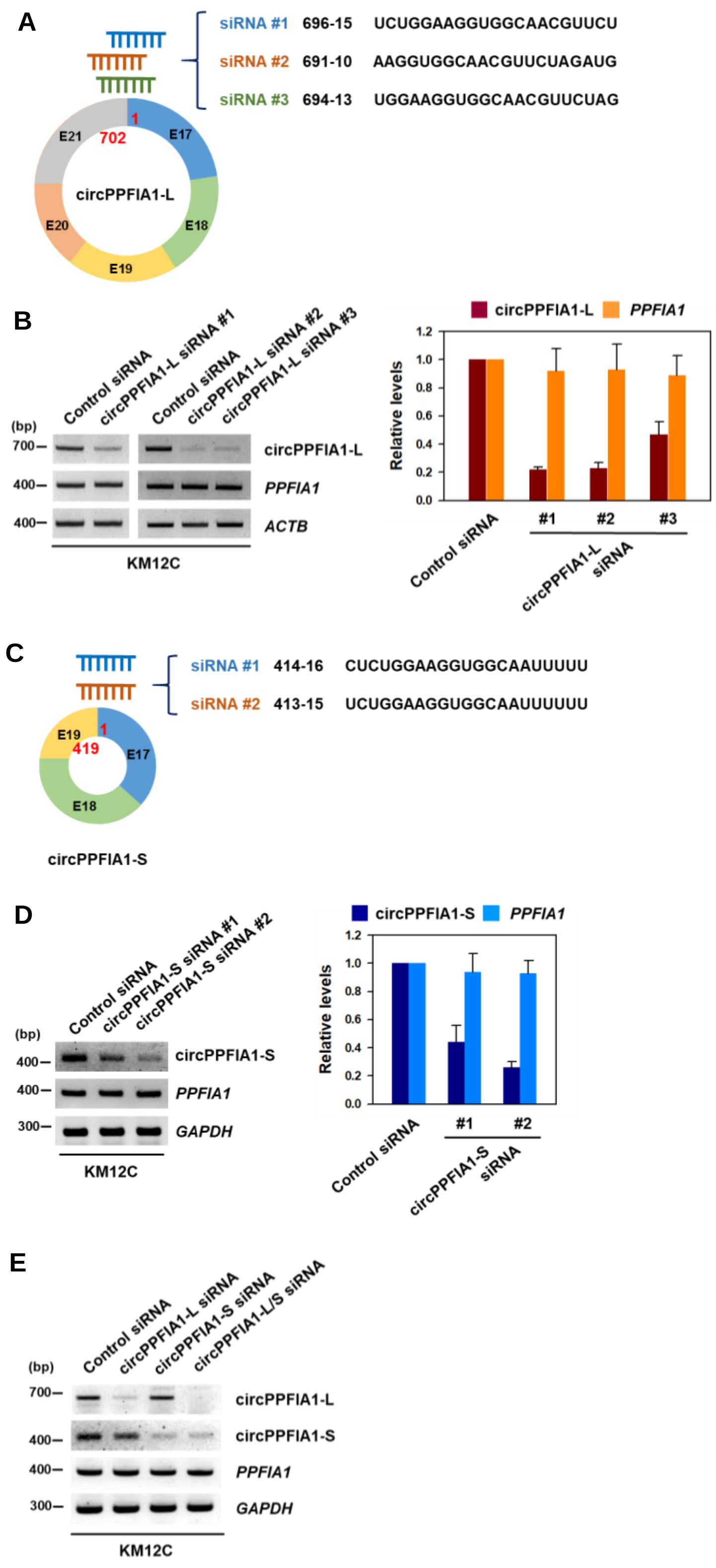
A
siRNA #1 696-15 UCUGGAAGGUGGCAACGUUCU
siRNA #2 691-10 AAGGUGGCAACGUUCUAGAUG
siRNA #3 694-13 UGGAAGGUGGCAACGUUCUAG
1
702
E17
E18
E19
E20
E21
circPPFIA1-L
B
Control siRNA
circPPFIA1-L siRNA #1
Control siRNA
circPPFIA1-L siRNA #2
circPPFIA1-L siRNA #3
(bp)
700
400
400
circPPFIA1-L
PPFIA1
ACTB
KM12C
circPPFIA1-L
PPFIA1
Relative levels
1.2
1.0
0.8
0.6
0.4
0.2
0.0
Control siRNA
#1
#2
#3
circPPFIA1-L siRNA
C
siRNA #1 414-16 CUCUGGAAGGUGGCAAUUUUU
siRNA #2 413-15 UCUGGAAGGUGGCAAUUUUUU
1
419
E17
E18
E19
circPPFIA1-S
D
Control siRNA
circPPFIA1-S siRNA #1
circPPFIA1-S siRNA #2
(bp)
400
400
300
circPPFIA1-S
PPFIA1
GAPDH
KM12C
circPPFIA1-S
PPFIA1
Relative levels
1.2
1.0
0.8
0.6
0.4
0.2
0.0
Control siRNA
#1
#2
circPPFIA1-S siRNA
E
Control siRNA
circPPFIA1-L siRNA
circPPFIA1-S siRNA
circPPFIA1-L/S siRNA
(bp)
700
400
400
300
circPPFIA1-L
circPPFIA1-S
PPFIA1
GAPDH
KM12C

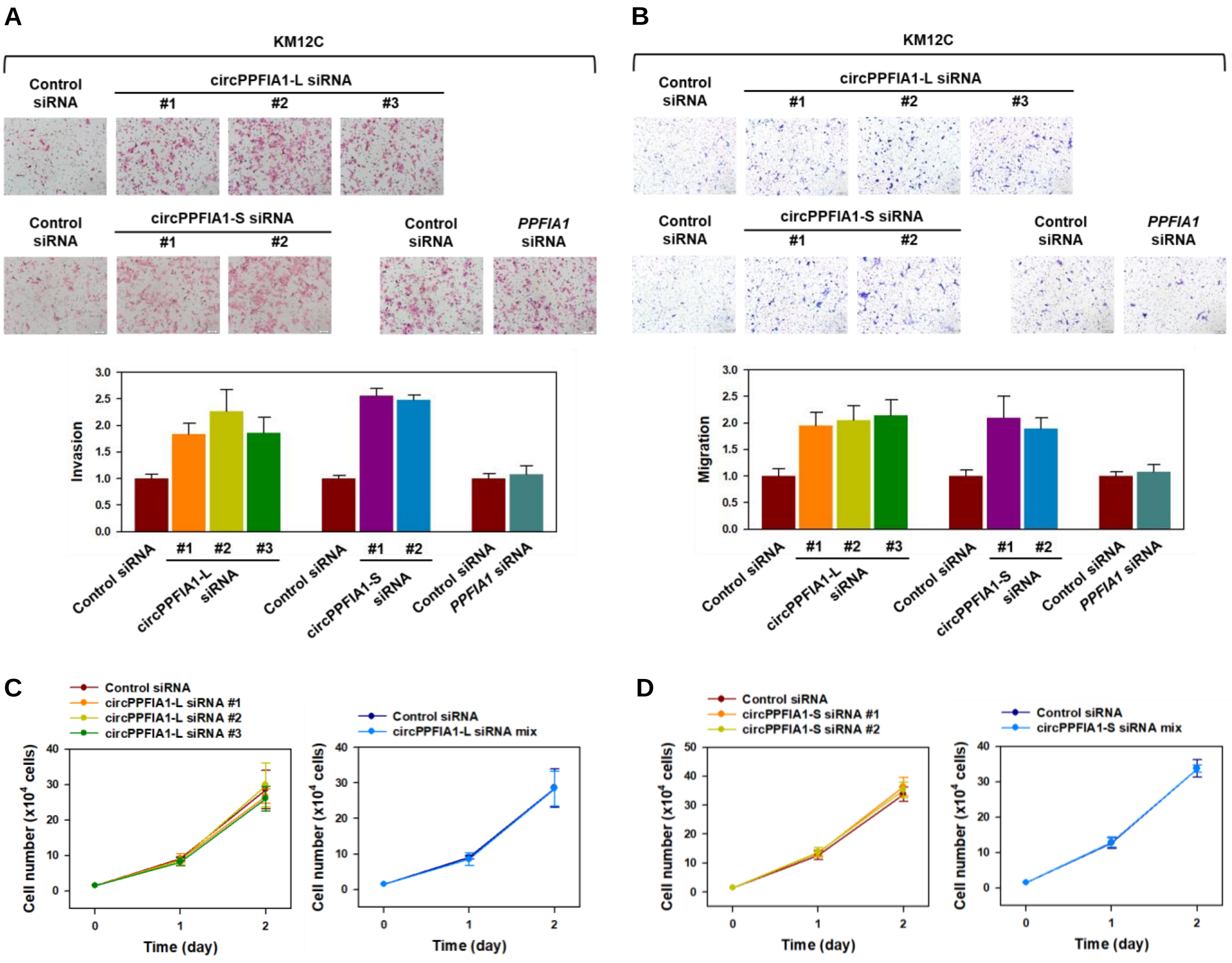
A
KM12C
Control siRNA
circPPFIA1-L siRNA
#1
#2
#3
circPPFIA1-S siRNA
PPFIA1 siRNA
Invasion
B
Migration
C
circPPFIA1-L siRNA #1
circPPFIA1-L siRNA #2
circPPFIA1-L siRNA #3
circPPFIA1-L siRNA mix
Cell number (x10^4 cells)
Time (day)
D
circPPFIA1-S siRNA #1
circPPFIA1-S siRNA #2
circPPFIA1-S siRNA mix

Supplementary Figure S9

Ji et al.

A

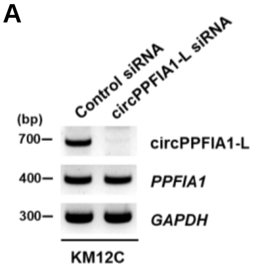

B

	1	2	3	4	5	6	7
Control siRNA *Incidence=57.1% (4/7)*	+	+	-	++	+	-	-
circPPFIA1-L siRNA *Incidence=100% (7/7)*	++	++	+	++	++	+	+++

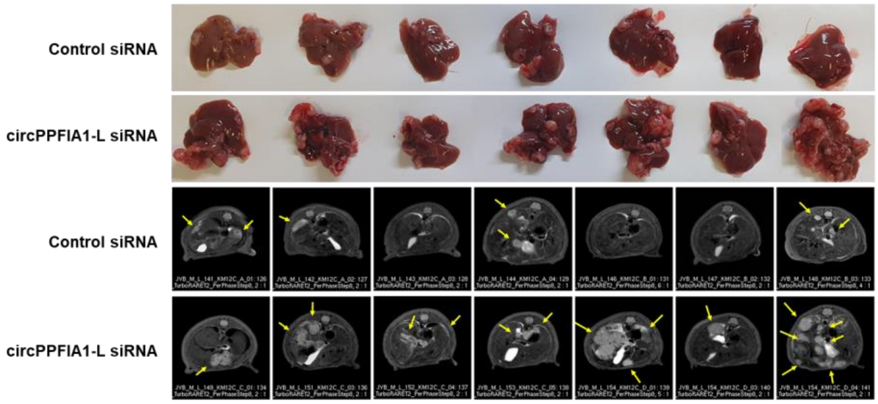

C

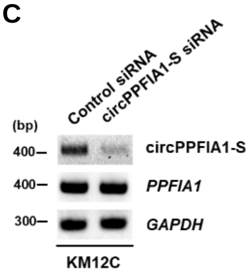

D

	1	2	3	4	5	6	7
Control siRNA *Incidence=28.6% (2/7)*	-	-	-	-	+	+	-
circPPFIA1-S siRNA *Incidence=100% (7/7)*	+++	+++	+++	+++	+++	++	++

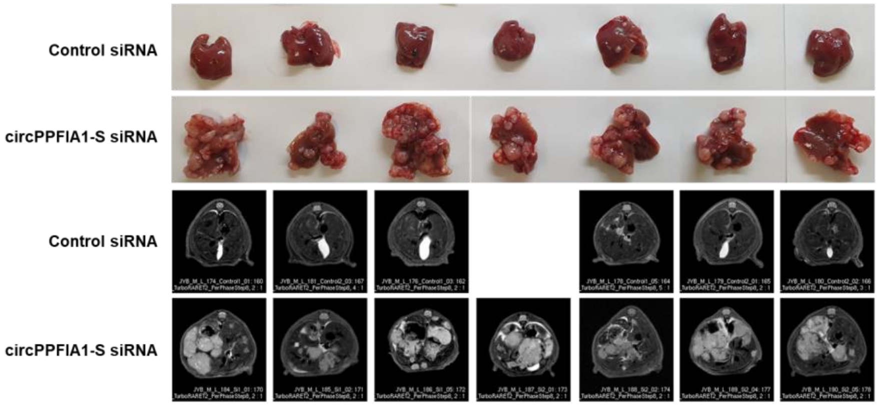

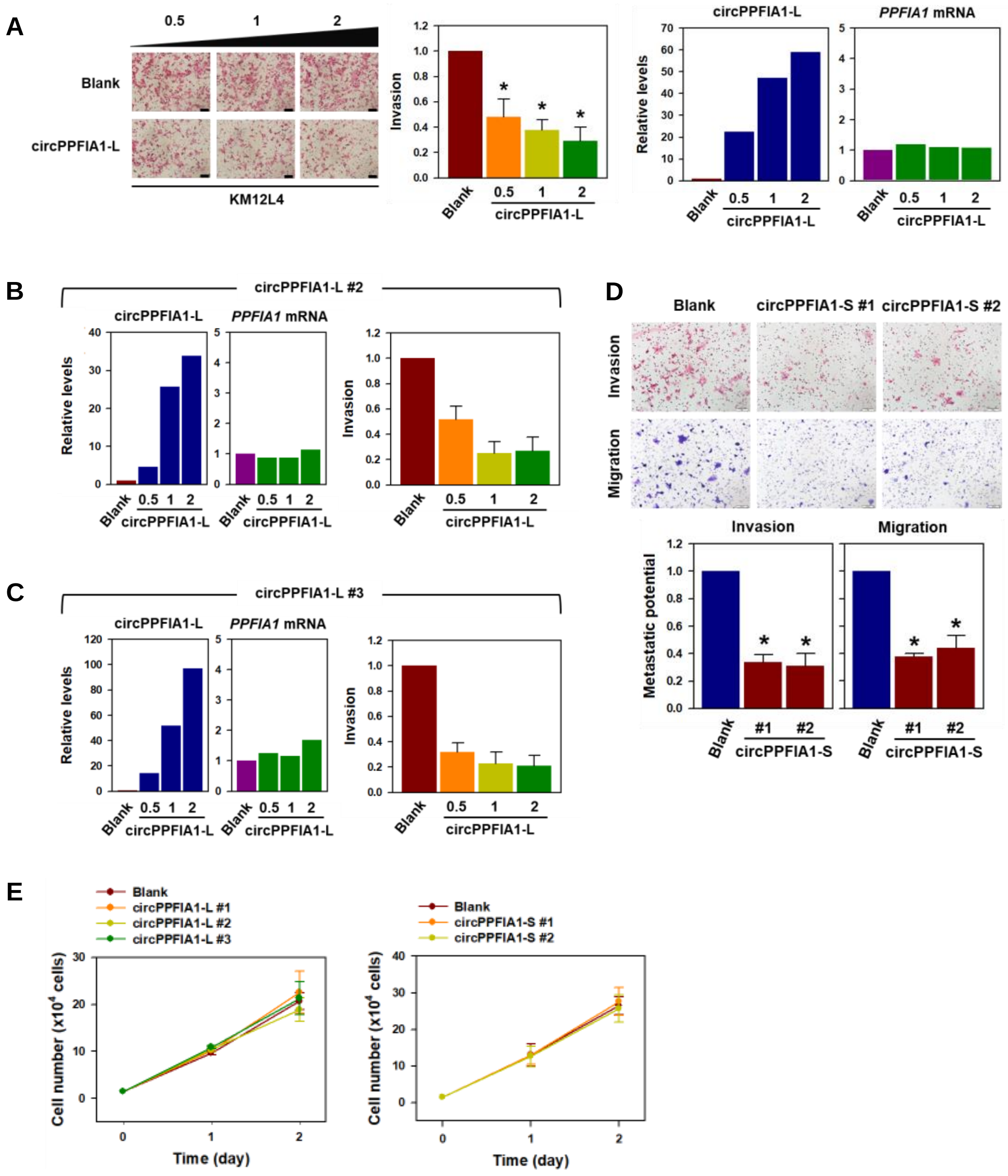
A
0.5
1
2
Blank
circPPFIA1-L
KM12L4
Invasion
1.2
1.0
0.8
0.6
0.4
0.2
0.0
*
Blank
0.5
1
2
circPPFIA1-L
circPPFIA1-L
PPFIA1 mRNA
Relative levels
70
60
50
40
30
20
10
0
5
4
3
2
1
B
circPPFIA1-L #2
circPPFIA1-L
PPFIA1 mRNA
Relative levels
40
30
20
10
Invasion
C
circPPFIA1-L #3
120
100
80
60
40
20
D
Blank
circPPFIA1-S #1
circPPFIA1-S #2
Invasion
Migration
Metastatic potential
#1
#2
circPPFIA1-S
E
circPPFIA1-L #1
circPPFIA1-L #2
circPPFIA1-L #3
Cell number (x10⁴ cells)
Time (day)
circPPFIA1-S #1
circPPFIA1-S #2

A

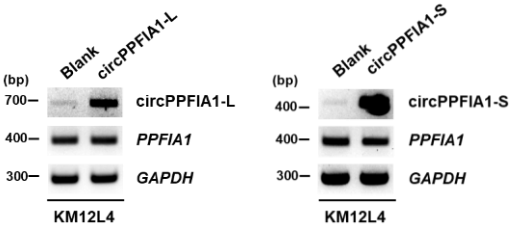

B

	1	2	3	4	5
Blank *Incidence=100% (5/5)*	+	++	+++	++	+
circPPFIA1-S *Incidence=60% (3/5)*	+	-	-	+	++
circPPFIA1-L *Incidence=80% (4/5)*	+	+	+	-	+

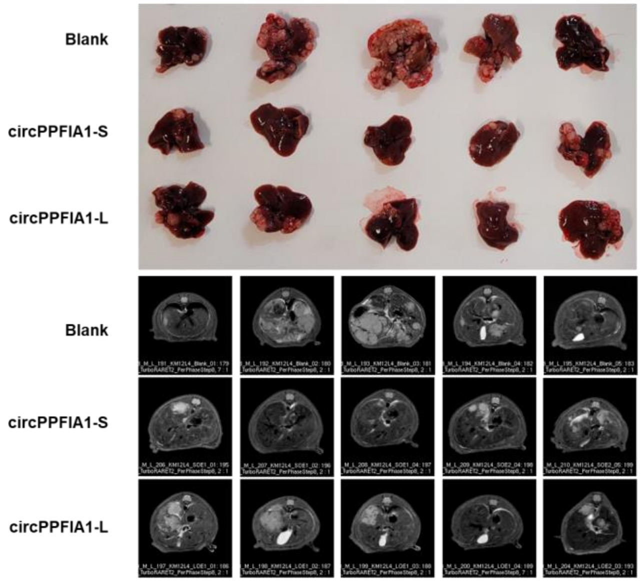

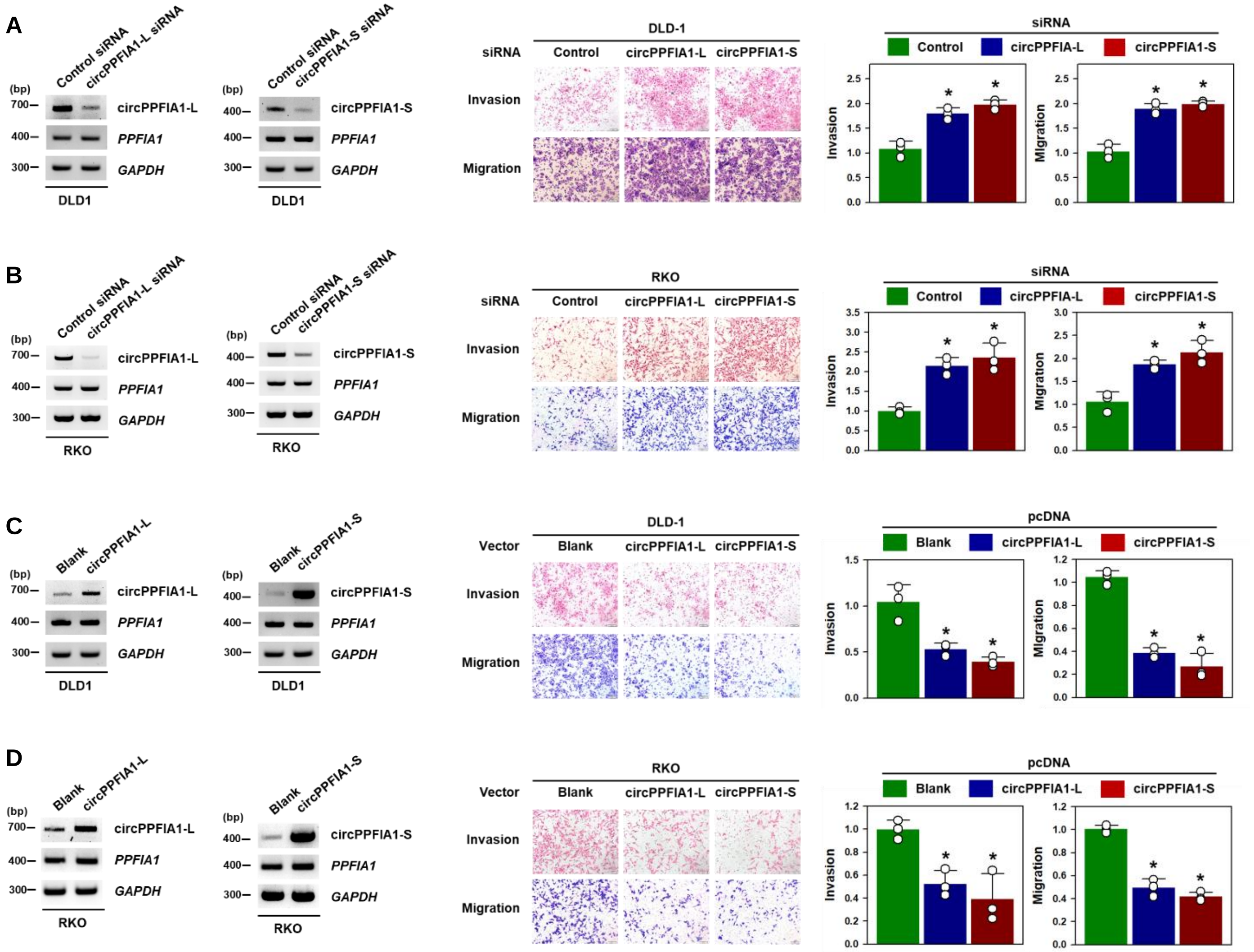
A
Control siRNA
circPPFIA1-L siRNA
circPPFIA1-S siRNA
(bp)
700
400
300
circPPFIA1-L
circPPFIA1-S
PPFIA1
GAPDH
DLD1
DLD-1
siRNA
Control
circPPFIA1-L
circPPFIA1-S
Invasion
Migration
Control
circPPFIA-L
circPPFIA1-S
B
RKO
C
Blank
Vector
pcDNA
circPPFIA1-L
circPPFIA1-S
D

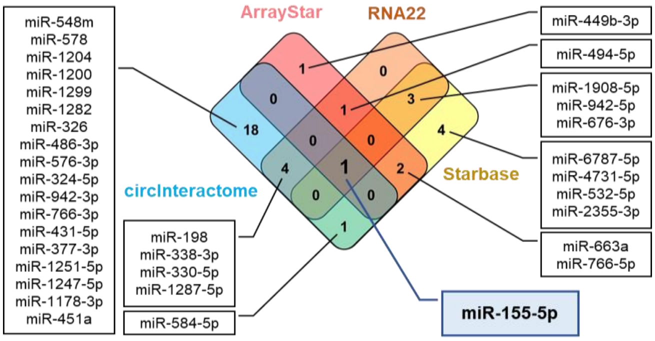
ArrayStar
RNA22
circInteractome
Starbase
miR-548m
miR-578
miR-1204
miR-1200
miR-1299
miR-1282
miR-326
miR-486-3p
miR-576-3p
miR-324-5p
miR-942-3p
miR-766-3p
miR-431-5p
miR-377-3p
miR-1251-5p
miR-1247-5p
miR-1178-3p
miR-451a
miR-198
miR-338-3p
miR-330-5p
miR-1287-5p
miR-584-5p
miR-449b-3p
miR-494-5p
miR-1908-5p
miR-942-5p
miR-676-3p
miR-6787-5p
miR-4731-5p
miR-532-5p
miR-2355-3p
miR-663a
miR-766-5p
miR-155-5p
1
0
0
3
18
1
4
0
0
2
4
1
0
0
1

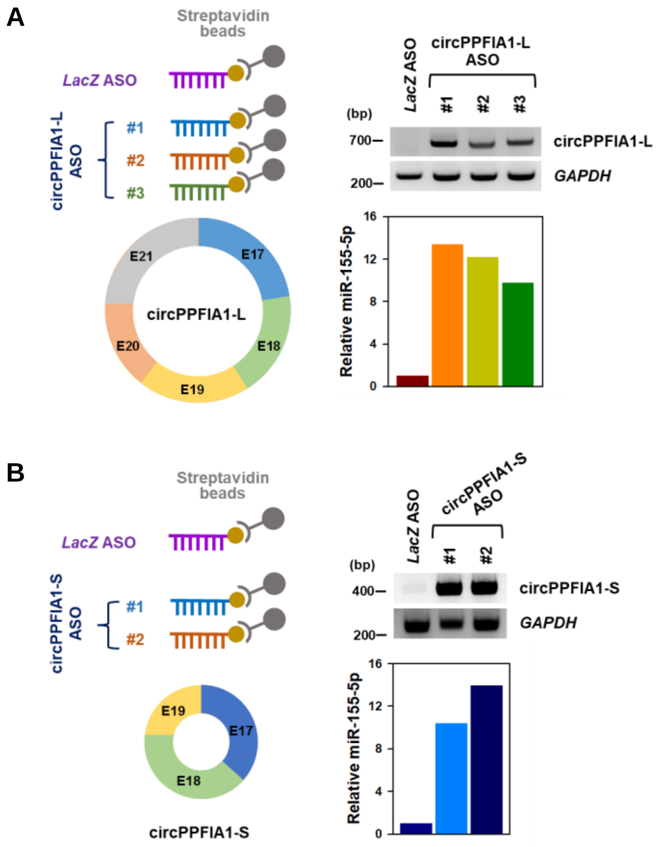

C

Sequences of antisense oligonucleotide (ASO)	
LacZ	GGGACGCGCGAAUUGAAUUA
circPPFIA1-L #1	UCUGGAAGGUGGCAACGUUCU
circPPFIA1-L #2	AAGGUGGCAACGUUCUAGAUG
circPPFIA1-L #3	UGGAAGGUGGCAACGUUCUAG
circPPFIA1-S #1	CUCUGGAAGGUGGCAAUUUUU
circPPFIA1-S #2	UCUGGAAGGUGGCAAUUUUUU

A

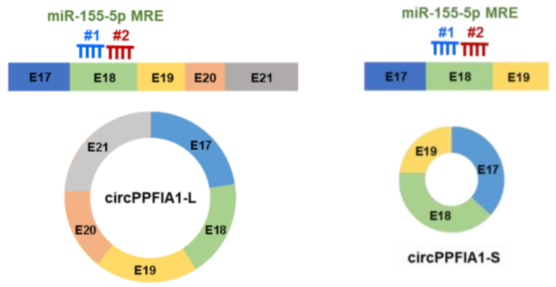
miR-155-5p MRE
#1 #2
E17 E18 E19 E20 E21
E21 E17 circPPFIA1-L E20 E18 E19
miR-155-5p MRE
#1 #2
E17 E18 E19
E19 E17 E18
circPPFIA1-S

B

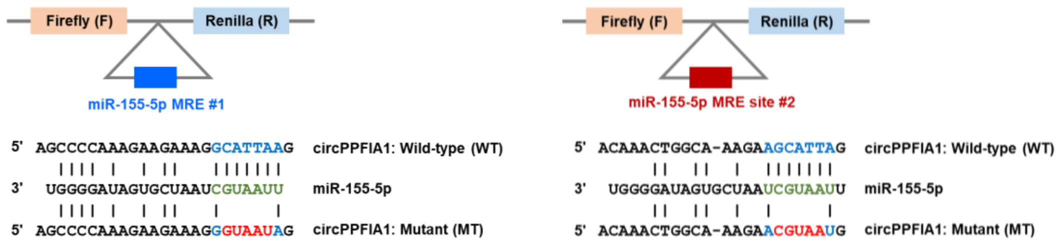
Firefly (F) Renilla (R)
miR-155-5p MRE #1
5' AGCCCCAAAGAAGAAAGGCATTAAG circPPFIA1: Wild-type (WT)
3' UGGGGAUAGUGCUAAUCGUAAUU miR-155-5p
5' AGCCCCAAAGAAGAAAGGGUAAUAG circPPFIA1: Mutant (MT)
Firefly (F) Renilla (R)
miR-155-5p MRE site #2
5' ACAAACTGGCA-AAGAAGCATTAG circPPFIA1: Wild-type (WT)
3' UGGGGAUAGUGCUAAUCGUAAUU miR-155-5p
5' ACAAACTGGCA-AAGAACGUAAUG circPPFIA1: Mutant (MT)

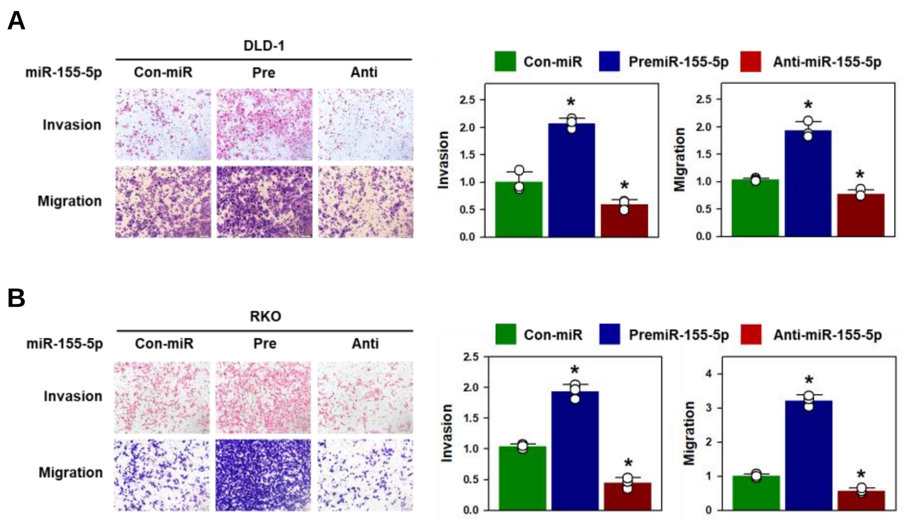
A
DLD-1
miR-155-5p
Con-miR
Pre
Anti
Invasion
Migration
Con-miR
PremiR-155-5p
Anti-miR-155-5p
Invasion
Migration
B
RKO
miR-155-5p
Con-miR
Pre
Anti
Invasion
Migration
Con-miR
PremiR-155-5p
Anti-miR-155-5p
Invasion
Migration

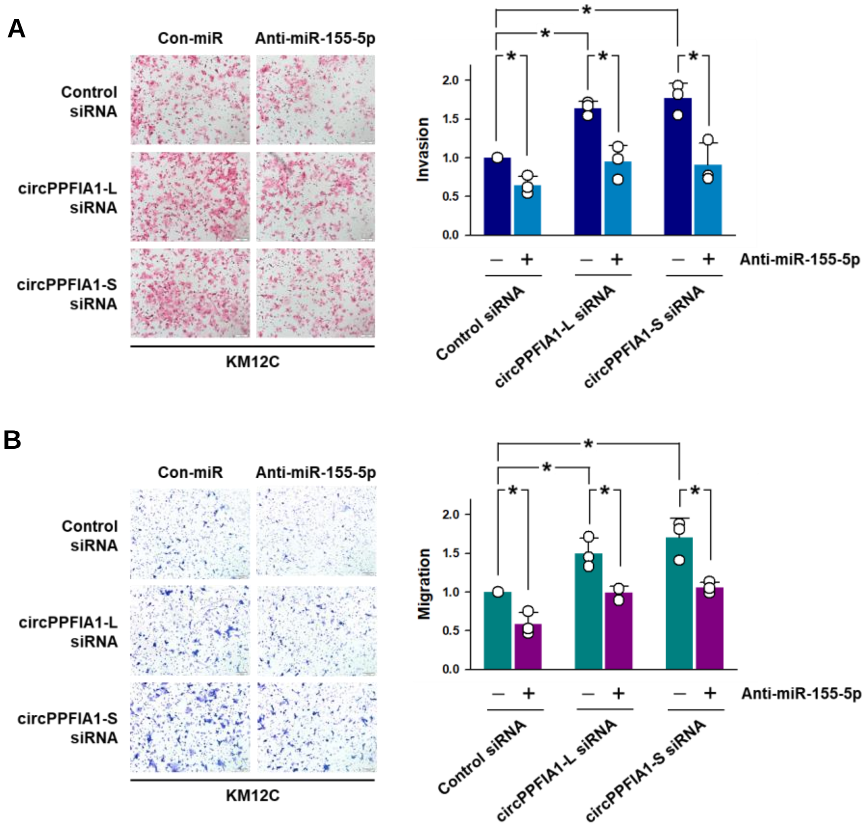
A
Con-miR
Anti-miR-155-5p
Control siRNA
circPPFIA1-L siRNA
circPPFIA1-S siRNA
KM12C
Invasion
2.0
1.5
1.0
0.5
0.0
*
Anti-miR-155-5p
Control siRNA
circPPFIA1-L siRNA
circPPFIA1-S siRNA
B
Con-miR
Anti-miR-155-5p
Control siRNA
circPPFIA1-L siRNA
circPPFIA1-S siRNA
KM12C
Migration
2.0
1.5
1.0
0.5
0.0
*
Anti-miR-155-5p
Control siRNA
circPPFIA1-L siRNA
circPPFIA1-S siRNA

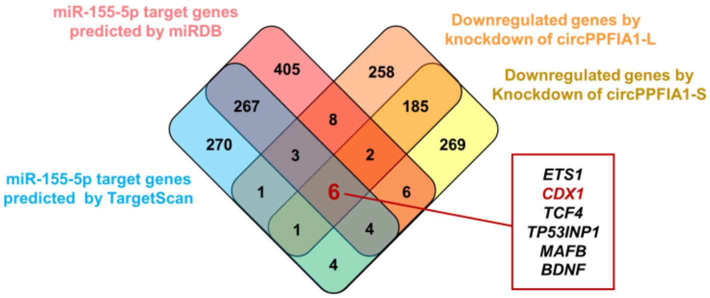
miR-155-5p target genes predicted by miRDB
Downregulated genes by knockdown of circPPFIA1-L
Downregulated genes by Knockdown of circPPFIA1-S
miR-155-5p target genes predicted by TargetScan
405
258
267
185
8
270
3
2
269
1
6
6
1
4
4
ETS1
CDX1
TCF4
TP53INP1
MAFB
BDNF

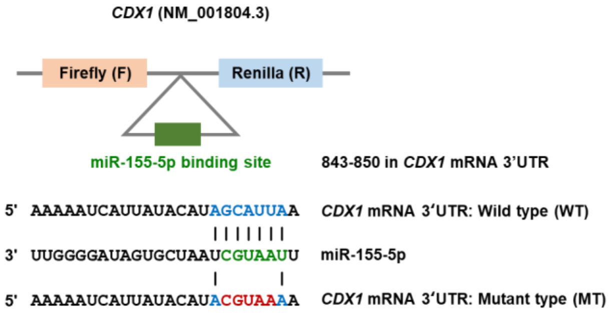
CDX1 (NM_001804.3)
Firefly (F)
Renilla (R)
miR-155-5p binding site
843-850 in CDX1 mRNA 3'UTR
5' AAAAAUCAUUAUACAUAGCAUUAA
CDX1 mRNA 3'UTR: Wild type (WT)
3' UUGGGGAUAGUGCUAAUCGUAAUU
miR-155-5p
5' AAAAAUCAUUAUACAUACGUAAAA
CDX1 mRNA 3'UTR: Mutant type (MT)

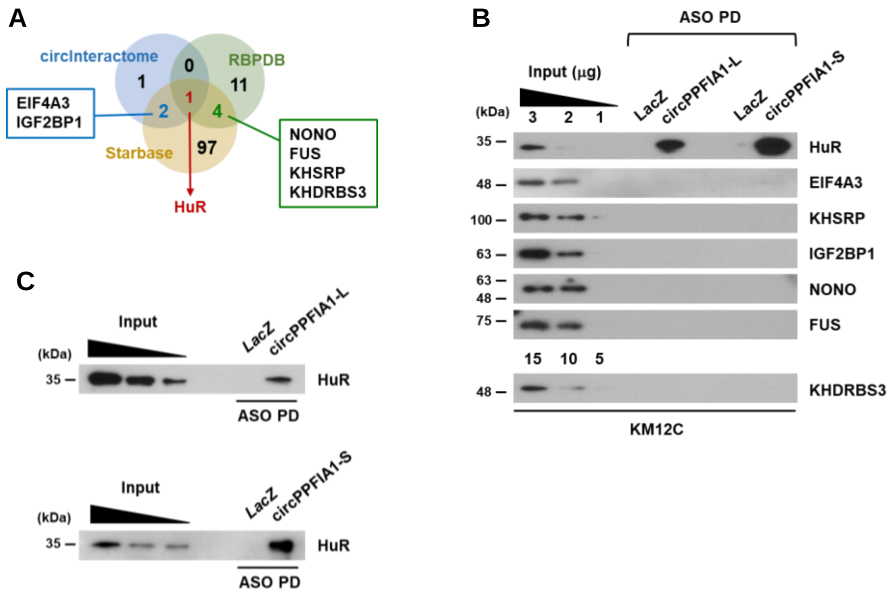
A
circInteractome
RBPDB
Starbase
1
0
11
2
1
4
97
EIF4A3
IGF2BP1
NONO
FUS
KHSRP
KHDRBS3
HuR
B
ASO PD
Input (µg)
LacZ
circPPFIA1-L
LacZ
circPPFIA1-S
(kDa)
3
2
1
35
HuR
48
EIF4A3
100
KHSRP
63
IGF2BP1
63
48
NONO
75
FUS
15
10
5
48
KHDRBS3
KM12C
C
Input
LacZ
circPPFIA1-L
(kDa)
35
HuR
ASO PD
Input
LacZ
circPPFIA1-S
(kDa)
35
HuR
ASO PD

Upregulated genes (>1.5): 62 genes

circPPFIA1-L siRNA *vs.* control siRNA
circPPFIA1-S siRNA *vs.* control siRNA
KM12L4 vs. KM12C

APLN	MT1F
ARMC4P1	NDP
CEMP1	RASSF10
CITED4	SLITRK6
CLDN22	TRIL
FBLL1	CCDC160
MORN3	GJD3

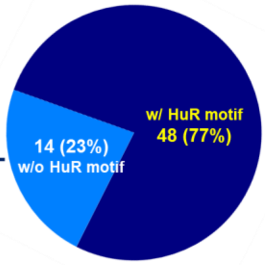

ACTL8	FOXN4	LMCD1	SAMD15
ADGRA2	FSIP1	LRGUK	SKIDA1
APOM	GNA14	LRRC73	SLC25A53
ATP13A4	GP2	MAP4K1	SMOC2
CAMK2N2	GPC3	MT1X	SPATA12
CARD9	GRAMD1A	NAPSA	SPIB
CD27	GRAMD2A	PAPLN	TINAG
CD74	HEATR4	PAX5	TMEM200B
CD8A	IGDCC4	PCDH7	TMEM240
CDK3	KIAA1324	**RAB36**	TNFSF13
CELA3A	LHX3	RFPL2	TSNAXIP1
DSG1	LHX9	RGS9BP	UFSP1

Supplementary Table S1. Verification of cell lines used in this study by STR analysis

Cell line		KM12C		KM12L4		DLD1		RKO	
Species		Human		Human		Human		Human	
STR		Ref	Obs	Ref	Obs	Ref	Obs	Ref	Obs
	AMEL	X	X	X	X	X,Y	X,Y	X	X
	CSF1PO	10	10,12	10,12	10,12	11,12	11,12	8,10	8,10
	D13S317	10	10	11	11	8,11	8,11	8,11	8,11
	D16S539	8	8	8,9	8	12,13	12,13	12,13	12,13
	D21S11	12,15	12,15	13,15	13,15	29,32,2	29,32,2	27,30	27,30
	D5S818	11	11	11,12	11,12	13	13	11,13,15	11,13,15
	D7S820	27,34.2	27,34.2	27,34.2	27,34.2	10,12	10,12	8,10	8,10
	TH01	10	9.3	10	9.3	7,9,3	7,9,3	6,10	6,10
	TPOX	11,12	11,12	11,12	11,12	8,11	8,11	11	11
	vWA	17	16,17	16,17	16,17	18,19	18,19	15,16,17,22	16,22
	Result	100		100		100		100	

Supplementary Table S2. PCR primers and siRNAs used in this study

Gene			Cat No. or sequence	company
RAB36	siRNA		9609-1 (Human, RAB36)	Bioneer, KR
	Primer		P241811	
CDX1	siRNA		1044-1 (Human, CDX1)	Bioneer, KR
	Primer		P310942	
GAPDH	RT-qPCR	Forward	TGCACCACCAACTGCTTAGC	
		Reverse	GGCATGGACTGTGGTCATGAG	
	Semi-PCR	Forward	CTGACTTCAACAGCGACACC	
		Reverse	AGGGGAGATTCAGTGTGGTG	
	Conventional	Forward	ACCCAGAAGACTGTGGATGG	
		Reverse	TGACAAAGTGGTCGTTGAGG	
	Divergent	Forward	TGAATCTCCCCTCCTCACAG	
		Reverse	TGCTGTAGCCAAATTCGTTG	
ACTB	Semi-PCR	Forward	AGAAAATCTGGCACCACACC	
		Reverse	CTCCTTAATGTCACGCACGA	

Supplementary Table S3. List of antibodies used in this study

Protein	Company	Cat No.
Ago2	Sigma-Aldrich	SAB4200085
RAB36	abcam	ab191531
CDX1	Santa Cruz	sc-515146
HuR	Santa Cruz	sc-5261
GAPDH	Santa Cruz	sc-47724
β-Actin	Sigma-Aldrich	A5441
α-Tubulin	Santa Cruz	sc-32293
Lamin B	Santa Cruz	sc-374015

Supplementary Table S4. Composition of buffers used in this study

RIPA	RSB	NT2	PEB	2X TENT
10 mM Tris-HCl, pH 7.4	10 mM Tris-HCl, pH 7.4	50 mM Tris-HCl, pH 7.4	20 mM Tris-HCl, pH 7.4	20 mM Tris-HCl, pH 8.0
1% Nonidet P-40	2.5 mM $MgCl_2$	0.05% Nonidet P-40	0.5% Nonidet P-40	2 mM EDTA
1 mM EDTA	100 mM NaCl	1 mM $MgCl_2$	100 mM KCl	500 mM NaCl
0.1% SDS		150 mM NaCl	5 mM $MgCl_2$	1% TritonX-100
150 mM NaCl				

Two circPPFIA1s negatively regulate liver metastasis of colon cancer via miR-155-5p/CDX1 and HuR/RAB36

■ Supplementary methods

Cell culture and transfection

Primary and liver metastatic CRC cells (KM12C and KM12L4, respectively) and colorectal cancer cells (DLD1 and RKO) were cultured with Dulbecco's modified Eagle's medium (Gibco, Grand Island, NY, USA) supplemented with 10% fetal bovine serum (Gibco) and 1% antibiotic-antimycotic solution (Gibco) in a 37°C incubator with 5% CO_2. All cell lines were free of mycoplasma contamination and verified by STR analysis (Supplementary Table S1). Cells were transfected with small interfering RNAs (siRNAs) and miRNAs using Lipofectamine 2000 (Invitrogen, Thermo Fisher Scientific, Waltham, MA, USA) according to the manufacturer's protocol. The control siRNAs for circPPFIA1-L and circPPFIA1-S, were synthesized by Bioneer (Daejeon, South Korea: Supplementary Figure S7). The precursor miR-155-5p (pre-miR-155-5p, PM13058) and antisense miR-155-5p (anti-miR-155-5p, AM13058) were purchased from Ambion (Ambion, Thermo Fisher Scientific). The predesigned siRNAs for the knockdown of CDX1 and RAB36 were purchased from Bioneer (Supplementary Table S2). For circPPFIA1 overexpression, the full length of human circPPFIA1-L and -S was synthesized and cloned into a pcDNA3.1(+) circRNA Mini Vector (No. 60648, Addgene, Watertown, MA, USA). The empty vector was used as a negative control.

Characterization of circRNA

The circular structure of circPPFIA1-L and -S was confirmed by testing the stability

via RNase R resistance and actinomycin D treatment. For the RNase R resistance test, total RNA was prepared as described above, and 1 μg of RNA was incubated with 2 U RNase R (Epicentre Technologies, Madison, WI, USA) at 37°C for 15 min. The remaining level of circPPFIA1s was determined by semi-qPCR and RT-qPCR. To examine the stability of circPPFIA1-L and -S, cells were treated with actinomycin D (5 μg/μl) and harvested at the indicated times. RNA was isolated, and the level of circPPFIA1-L and -S was determined by semi-qPCR and RT-qPCR. Linear *PPFIA1*, *GAPDH*, and *ACTB* mRNA were used for control. Information on PCR primers is presented in Supplementary Figure S4 and Table S2. For Sanger sequencing, KM12C cDNA was amplified by semi-quantitative PCR as described above with the appropriate primers and the product was purified using the AccuPrep® PCR/Gel Purification Kit (Bioneer, South Korea). Sanger sequencing was performed by Bioneer.

Western blot analysis

Cells were washed with ice-cold PBS and lysed with RIPA buffer containing an inhibitor mixture of protease and phosphatase (Roche, Basel, Switzerland). Equal amount of lysates were separated using sodium dodecyl sulfate-polyacrylamide gel electrophoresis (SDS-PAGE) and were transferred to a polyvinylidene difluoride (PVDF) membrane (Millipore Sigma, Burlington, MA, USA). The membranes were incubated with the indicated primary antibody at 4°C overnight, followed by incubation with the appropriate secondary antibody. GAPDH or β-Actin were used as a loading control. Detailed information on antibodies is presented in Supplementary Table S3.

Semi-quantitative (semi-qPCR) and real-time quantitative polymerase chain reaction (RT-qPCR) analyses

Total RNA was isolated using TRIzol reagent (Invitrogen, Thermo Fisher Scientific) according to the manufacturer’s protocol. To determine the expression level of circRNA and mRNA, 1 μg of RNA was used as a template to synthesize complementary DNA (cDNA) using

the SuperScript III First-Strand Synthesis System (Invitrogen, Thermo Fisher Scientific). The cDNA was subjected to RT-qPCR using Power SYBR Green PCR Master Mix (Applied Biosystems, Foster City, CA, USA) with the appropriate primers. *GAPDH* was used as an internal control. To measure the miR-155-5p level, the miRNA-specific TaqMan primer (#4427975, ID:002623, Applied Biosystems, Thermo Fisher Scientific) was used for RT-qPCR. For semi-qPCR analysis, cDNA was subjected to semi-quantitative PCR (ProFlex PCR system, Applied Biosystems) using AccuPower® HotStart Pfu Premix (Bioneer) with the appropriate primers. Amplified PCR products were electrophoresed on a 2% agarose gel (Biosesang, South Korea) and the intensity of the bands was calculated using ImageJ software (National Institutes of Health, Bethesda, MD, USA). The primer sequences for semi-qPCR and RT-qPCR are listed in Supplementary Figure S4 and Table S2.

Transwell migration and invasion assays

To examine invasive and migratory abilities, transwell assays were performed using a transwell chamber (Corning, NY, USA). An equal number of cells was seeded into the upper chamber with serum-free media, and 750 μl of media supplemented with 10% FBS was added to the lower chambers as a chemo-attractant. After 24 h, the cells were fixed with 95% MeOH for 5 min and stained with 0.1% hematoxylin and eosin. The metastatic potential was determined by counting the number of invaded and migrated cells in at least ten randomly selected fields.

Luciferase reporter assay

To verify direct interactions between miR-155-5p and circPPFIA1, KM12C cells were transfected with control miRNA or pre-miR-155-5p. After incubation for 24 h, mirGLO dual-luciferase vectors (E133A, Promega, Madison, WI, USA) containing wild-type or mutant miR-155-5p miRNA response element (MRE) sequences from circPPFIA1 were introduced into the cells. Luciferase activity was assessed using the Dual-GLO™ Luciferase Activity Assay

System (E2940, Promega).

■ Supplementary figures legends

Supplementary Figure S1. Comparison of metastatic potential of primary (KM12C) and liver metastatic colorectal cancer cells (KM12L4). (**A**) Schematic illustration of the establishment of the liver metastatic colorectal cancer cell model. KM12C was originally obtained from a human specimen and KM12L4 was generated through the fourth selection-isolation of intrasplenic injection. (**B**) Liver metastases were examined using in vivo intra-splenic injection. At four weeks post-injection, optical and MRI images were obtained. The degree of liver metastasis (n = 5) was calculated by giving scores in arbitrary units (0–3).

Supplementary Figure S2. Analyses of circRNA microarray data. (**A**) A heat map analysis of circRNA microarray. (**B**) A volcano plot of circRNA microarray data showing a list of the upregulated and downregulated circRNAs. (**C**) Detailed information on circPPFIA1-L and -S.

Supplementary Figure S3. List of *PPFIA1*-originated circRNAs. circRNAs that are generated from the *PPFIA1* gene are listed in a public circRNA database (circBase, http://www.circbase.org).

Supplementary Figure S4. Primer sequences used in this study. (**A**) Schematic illustration and sequences of RT-qPCR primers detecting circPPFIA1-L, circPPFIA1-S, and linear *PPFIA1*. (**B**) Validation of the expression of circPPFIA1-L and -S by RT-qPCR using the above primer sets. (**C**) Schematic illustration and sequences of semi-qPCR primers detecting circPPFIA1-L, circPPFIA1-S, and linear *PPFIA1*. (**D**) Schematic illustration and sequences of primers for Sanger sequencing.

Supplementary Figure S5. Comparison of circPPFIA1-L and -S expression between adjacent normal and tumor tissues of CRC patients. Tumor tissues and their matched

normal tissues were obtained from 14 CRC patients at the Samsung Medical Center. Among the 14 patients, seven did not exhibit metastasis while the other seven patients showed liver metastasis. All samples were collected with the informed consent of patients under institutional review board-approved protocols and stored at -80°C until use. The expression level of circPPFIA1-L and -S was determined by RT-qPCR (n = 38 for circPPFIA1-L, n = 28 for circPPFIA1-S).

Supplementary Figure S6. Characterization of circPPFIA1-L and -S. (**A**) The stability of circPPFIA1s and linear *PPFIA1* was examined by RT-qPCR using total RNA isolated from actinomycin D-treated KM12C cells. (**B**) Schematic illustration of divergent and convergent semi-qPCR primers for circPPFIA1-L and -S. Semi-qPCR analysis was conducted using genomic (gDNA) and complementary (cDNA) DNA. *GAPDH* was used as a negative control. (**C**) Sanger sequencing results of circPPFIA1-L and -S.

Supplementary Figure S7. Design and validation of siRNAs targeting circPPFIA1-L and -S. (**A**, **C**) Schematic illustration and sequences of circPPFIA1-L siRNAs (A), and -S (C). (**B**, **D**) Each siRNA efficiently decreases the expression level of circPPFIA1-L (B) and -S (D). (**E**) For a cotransfection experiment, KM12C cells were simultaneously transfected with siRNAs targeting circPPFIA1-L and -S. The expression levels of circPPFIA1s and *GAPDH* were determined by semi-qPCR.

Supplementary Figure S8. Potentiation of metastatic properties by knockdown of circPPFIA1-L and -S in KM12C cells. (**A**, **B**) KM12C cells were transfected with the indicated siRNA (shown in Supplementary Figure S7), and transwell assays were performed to determine invasive (A) and migratory (B) abilities. (**C**, **D**) Cell proliferation of KM12C cells transfected with individual or a mixture of siRNAs targeting circPPFIA1-L (C) or -S (D) was determined by counting the number of viable cells.

Supplementary Figure S9. Increase in liver metastasis in vivo by knockdown of circPPFIA1s. The effect of circPPFIA1-L and -S on liver metastasis in vivo was examined through intrasplenic injection of KM12C cells transfected with circPPFIA1s siRNAs. (**A**, **C**) The expression level of circPPFIA1-L (A) and -S (C) was determined by semi-qPCR. (**B**, **D**) Liver metastases were examined using in vivo intrasplenic injection. At four weeks post-injection, optical and MRI images were obtained. The degree of liver metastasis (n = 7) was calculated by giving scores in arbitrary units (0–3).

Supplementary Figure S10. Suppression of metastatic potential by overexpression of circPPFIA1-L and -S in KM12L4 cells. (**A-C**) By introducing the circPPFIA1-L overexpression vector into KM12L4 cells, three independent clones (#1–#3) were generated and were used to investigate the effect of circPPFIA1-L on metastatic potential. Invasive ability was examined by transwell invasion assays, and the expression levels of circPPFIA1-L and *PPFIA1* mRNA were measured by RT-qPCR. (**D**) In the same way as above, two clones (#1 and #2) in which circPPFIA1-S was overexpressed were generated, and invasive and migratory abilities were determined by transwell invasion and migration assays. (**E**) The effect of circPPFIA1 overexpression on cell proliferation was examined by counting the number of viable cells.

Supplementary Figure S11. Decrease in liver metastasis in vivo by overexpression of circPPFIA1s. The effect of circPPFIA1-L and -S on liver metastasis in vivo was examined via intrasplenic injection of KM12L4 cells, wherein circPPFIA1-L or -S was overexpressed. (**A, C**) The expression levels of circPPFIA1-L (A) and -S (C) were determined by semi-qPCR. (**B**, **D**) Liver metastases were examined via in vivo intrasplenic injection of circPPFIA1-L (B) or -S (D) overexpressing KM12L4 cells. At four weeks post-injection, optical and MRI images were obtained. The degree of liver metastasis (n = 7) was calculated by giving scores in arbitrary

units (0–3).

Supplementary Figure S12. Negative regulation of invasive and migratory abilities via circPPFIA1-L and -S in DLD1 and RKO cells. (**A**, **B**) Following transfection of DLD1 (A) and RKO (B) cells with indicated siRNA, transwell assays were conducted to measure invasive and migratory abilities. The expression levels of circPPFIA1-L, -S, and *PPFIA1* mRNA were determined by semi-qPCR. *GAPDH* was used as a loading control. (**C**, **D**) For overexpression of circPPFIA1-L and -S, DLD1 (C) and RKO (D) cells were transfected with indicated vector. The number of invaded and migrated cells was assessed using transwell assays. The expression levels of circPPFIA1-L, -S, and *PPFIA1* mRNA were determined by semi-qPCR. *GAPDH* was used as a loading control.

Supplementary Figure S13. Venn diagram for selecting putative interacting miRNAs with circPPFIA1. By prediction of circPPFIA1-interacting miRNAs using four prediction algorithms (ArrayStar, RNA22, circInteractome, and Starbase), miR-155-5p was selected for further studies.

Supplementary Figure S14. Schematic illustration and design of antisense oligonucleotide (ASO) for the pulldown experiments. (**A**, **B**) ASOs for pulldown were designed to bind to the divergent region of circPPFIA1-L (A) or -S (B). circPPFIA1-L and -S were captured using three and two ASOs, respectively. The enrichment of the corresponding circRNA in pulldown materials was assessed by semi-qPCR. (**C**) The sequences of ASOs for pulldown experiments. *LacZ* was used as a control ASO.

Supplementary Figure S15. Construction of luciferase vectors harboring wild-type (WT) or mutant (MT) sequences of miR-155-5p miRNA recognition element (MRE) in circPPFIA1-L and -S. (**A**) Two miR-155-5p MREs (#1 and #2) were predicted by bioinformatic

approaches in the sequence of exon 18 of *PPFIA1*. (**B**) Dual-luciferase vectors harboring wild-type (WT) or mutant (MT) sequences of each miR-155-5p MRE were manufactured.

Supplementary Figure S16. Regulation of metastatic potential by miR-155-5p in DLD1 and RKO cells. DLD1 (**A**) and RKO (**B**) cells were transfected with pre-miR-155-5p or anti-miR-155-5p. Invasive and migratory abilities were assessed using transwell invasion and migration assays.

Supplementary Figure S17. Rescue experiment for proving that miR-155-5p is required for the increase in metastatic potential by knockdown of circPPFIA1. KM12C cells were simultaneously transfected with circPPFIA1 siRNA and anti-miR-155-5p. Invasive and migratory abilities were examined using transwell invasion (**A**) and migration (**B**) assays.

Supplementary Figure S18. Venn diagram for screening common target genes that are regulated via circPPIFIA1s/miR-155-5p. By comparing the lists of downregulated genes obtained from RNA sequencing data, and miR-155-5p target genes predicted by miRDB and TargetScan, six genes (ETS1, CDX1, TCF4, TP53INP1, MAFB, and BDNF) were identified as putative targets.

Supplementary Figure S19. Schematic of dual-luciferase reporter vectors harboring wild-type or mutant sequences of miR-155-5p MRE in *CDX1* mRNA.

Supplementary Figure S20. Prediction and validation of circPPFIA1-interacting RNA-binding proteins. (**A**) Venn diagram for selecting circPPFIA1-interacting RBPs using three prediction algorithms (circInteractome, Starbase, and RBPDB). (**B**) The levels of predicted RBPS in ASO pulldown materials were assessed by western blot analyses using the indicated antibodies. (**C**) ASO pulldown followed by western blot analysis was conducted to verify the

interaction of HuR with circPPFIA1s (left, circPPFIA1-L; right, circPPFIA1-S).

Supplementary Figure S21. High proportion of common target genes harbor the HuR-binding motif. Upregulated common target genes were screened based on the following three criteria: (i) genes upregulated genes by knockdown of circPPFIA1-L, (ii) genes upregulated genes by knockdown of circPPFIA1-S, (iii) genes upregulated in KM12L4 compared to KM12C. By comparing and analyzing the public data of HuR CLIP-seq with the selected common target genes, we observed that 77% (48 genes out of 62 genes) of selected target genes have HuR-binding motifs in their 3'-UTRs.

■ **Supplementary tables**

Supplementary Table S1. Verification of cell lines used in this study by STR analysis

Supplementary Table S2. PCR primers and siRNAs used in this study

Supplementary Table S3. List of antibodies used in this study

Supplementary Table S4. Composition of buffers used in this study
